# Associations between an obesity-related dietary pattern and incidence of overall and site-specific cancers: a prospective cohort study

**DOI:** 10.1186/s12916-023-02955-y

**Published:** 2023-07-10

**Authors:** Maiwulamujiang Maimaitiyiming, Hongxi Yang, Lihui Zhou, Xinyu Zhang, Qiliang Cai, Yaogang Wang

**Affiliations:** 1 School of Public Health, Tianjin Medical University, Qixiangtai Road 22, Heping District, Tianjin, 300070 China; 2 Department of Bioinformatics, School of Basic Medical Sciences, Tianjin Medical University, Tianjin, China; 3 Department of Urology, the Second Hospital of Tianjin Medical University, Tianjin Institute of Urology, Tianjin, China

**Keywords:** Dietary pattern, Cancer risk, Cohort study, Reduced-rank regression

## Abstract

**Background:**

A dietary pattern (DP) may impact on cancer incidence more strongly than individual foods, but this association remains uncertain. Here, we aimed to broadly explore the associations of an obesity-related DP with overall and 19 site-specific cancers.

**Methods:**

This study included 114,289 cancer-free participants with at least two dietary assessments. A total of 210 food items were classified into 47 food groups, and the mean amount of each food group was used in reduced-rank regression to derive the obesity-related DP. Cox regressions were conducted to explore the associations of the obesity-related DP with overall and 19 site-specific cancers. The parallel mediation model was constructed to quantify the mediating roles of potential mediators.

**Results:**

During a median follow-up period of 9.4 years, 10,145 (8.9%) incident cancer cases were documented. The derived-DP was characterized by a higher intake of beer and cider, processed meat, high sugar beverages, red meat, and artificial sweetener, and a lower intake of fresh vegetables, olive oil, tea, and high fiber breakfast cereals. Observational analysis showed that a higher obesity-related DP Z-score was linearly associated with an increased risk of overall cancer (adjusted hazard ratio (HR) = 1.02, 95% CI: 1.01, 1.04 per 1-SD increase, corrected *P* < 0.001). For site-specific cancer, positive linear associations for six cancer sites (oral, colorectal, liver, lung, endometrium, and thyroid) and nonlinear associations for six cancer sites (esophagus, malignant melanoma, prostate, kidney, bladder, and multiple myeloma) were observed. The paralleled mediation analysis suggested that the association between the obesity-related DP and overall cancer is mediated by the body mass index (BMI), the waist-to-hip ratio (WHR), C-reactive protein, high-density lipoproteins (HDLs), and triglycerides.

**Conclusions:**

The developed obesity-related DP is strongly associated with overall and multiple cancer sites. Our findings highlight the complicated and diverse associations between an obesity-related DP and cancers and provide clues for future research directions.

**Supplementary Information:**

The online version contains supplementary material available at 10.1186/s12916-023-02955-y.

## Background

Cancer produces poor health outcomes and imposes a heavy burden on society. Globally, cancer was responsible for 9.6 million deaths and 18.1 million cases in 2018 [1]. Therefore, there is an urgent need to reduce cancer incidence by altering modifiable risk factors, such as diet, one of the established determinants for several site-specific cancer [2]. Specific unhealthy dietary factors were identified as risk factors for corresponding cancer sites by the World Cancer Research Fund (WCRF) Third Expert Report [2]. Therefore, the relationship between diet and multiple site-specific cancer has been a persistent concern by the general population and researchers. Although numerous studies have investigated isolated nutrients and food, the health effect of individual dietary factors on cancer is thought to be limited and equivocal. By contrast, the exploration of dietary patterns (DPs) may yield stronger cancer-related health effects and more reliable estimations, so the results can be more readily applied on clinical practice and dietary guidelines [3, 4]. DPs can be developed based on investigator-defined criteria or data-driven analyses. For instance, we can classify food items into several subsets and assign them scores based on prior knowledge, or we can extract the DP by a data-driven technique such as reduced rank regression (RRR), which derives DPs with the help of the given response variables. Compared to methods such as principal component analysis and index-based methods, RRR combines prior knowledge with a posteriori data-driven technique. RRR uses disease-related information (prior knowledge) to select response variables, which are pre-hypothetical intermediates between DPs and diseases, and then empirically derives DPs (posterior data-driven technique) to explain the maximum variations in response variables. Therefore, it is more likely to develop DPs related to the given health outcomes. Moreover, RRR can test a putative hypothesis of disease pathophysiology [5], which is helpful for mechanistic evidence. In sum, we need to select a set of response variables on the pathway between diets and cancer to derive the DP.

Indeed, diets play various roles in different pathways such as weight gain [6], apoptosis [7], oxidative DNA damage [8], and interplay with gut microbiota [9]. Among these pathways, obesity, a pandemic metabolic disorder disease, is a vital and modifiable mediator between diets and cancer that rose from nearly 20% to 40% among adults from 1975 to 2016 [10]. The associations between obesity and overall and site-specific cancers have been validated by a mendelian randomization study for the UK Biobank [11] and other large cohort studies [12, 13]. Moreover, obesity can be evaluated in a non-invasive and low-cost way compared to other metabolic syndrome (MetS) components (except for blood pressure), which are assessed by blood samples. Hence, proxy indicators of obesity were the ideal response variables to derive the obesity-related DP for this study. Therefore, in the present study, we first developed an obesity-related DP employing RRR and then extensively examined the prospective associations of the obesity-related DP with overall cancer and 19 site-specific cancers in the general UK population.

## Methods

### Study design and population

We obtained data from UK Biobank, a large prospective cohort, and national health resource, which enrolled over half a million participants aged 40–69 years from the general population between 2006 and 2010. Participants were invited to 1 of 22 assessment centers across England, Scotland, and Wales. They completed touchscreen and nurse-led questionnaires**,** took the anthropometric measurements and provided blood samples [14]. In the present study, we excluded participants who had no (*n* = 291,785) or 1 time 24 h (*n* = 84,163) dietary assessment and a diagnosis of cancer (other than nonmelanoma skin cancer, based on the International Classification of Diseases, 10th Revision [ICD-10] code C44) before the last dietary assessment (*n* = 11,353), implausible energy intake [15] (*n* = 914, outside of the range of 500 -3500 kcal/d for women and 800–4200 kcal/d for men), withdrew authorization in the study time (*n* = 24), leaving data from 114,289 remaining participants to be included in this study.

### Dietary assessment

UK Biobank collected dietary data on up to 5 separate occasions containing baseline and four rounds online (Cycle 1: February to April in 2011; Cycle 2: June to September in 2011; Cycle 3: October to December in 2011; Cycle 4: April to June in 2012) with inviting participants who responded via e-mail. All respondents completed the 24-h online dietary assessment (Oxford WebQ), which has been validated to estimate nutrient intakes [16]. We classified more than 210 food items into 47 food groups based on their similarities in nutrient profiles and habitual culinary practices (Additional file 1**: **Table S1). Participants who completed 2 or more dietary assessments were selected to avoid contingency and reflect usual dietary intakes. Firstly, we computed the amounts of each food item based on a validated portion size table [17]. The total weight of food groups was calculated by summing the weight of food items according to pre-classified categories. We then calculated the average amount of each food group for further analysis. Moreover, Oxford WebQ showed acceptable reproducibility when at least two dietary assessments were conducted [18].

### Outcome ascertainment

In the present study, we set the date of the last dietary assessment as baseline time and ended follow-up until the date of the first diagnosis of cancer, or death, or loss to follow-up, or deadline of observation (2021–09-30), whichever occurred first. In these analyses, the outcomes assessed consisted of overall cancer (other than nonmelanoma skin cancer, C44) and 19 common cancer sites with the highest incidences [1], as follows: oral, esophagus, stomach, colorectal, liver, pancreas, lung, malignant melanoma, premenopausal and postmenopausal breast, cervix, endometrium, ovary, prostate, kidney, bladder, thyroid, non-Hodgkin lymphoma, multiple myeloma, and leukemia cancers. We defined these cancer sites by the ICD-10 (Additional file 1**: **Table S2).

### Covariates

We used BMI and waist-to-hip ratio as response variables that reflected the degree of general and central obesity. At baseline, specialized staff measured body weight, standing height, waist circumferences, and hip circumferences. The BMI and waist-to-hip ratio (WHR) values were calculated by dividing body weight (kilogram) by the square of body height (meter) and the waist circumference (centimeter) by the hip circumference (centimeter). The potential confounders of our analyses included sociodemographic, behavioral risk factors, and health conditions and treatments related to cancer morbidities. Sociodemographic variables included age, sex, ethnicity, study regions, Townsend deprivation index, and education attainment. Townsend deprivation index, a comprehensive indicator, could reflect housing, employment, social class, and car availability, higher index corresponds to more deprivation. Behavioral risk factors included smoking status, physical activity [Low physical activity (< 600 metabolic equivalents (MET)-minutes/week); Moderate physical activity (≥ 600 and < 3,000 MET-minutes/week); High physical activity (≥ 3,000 MET-minutes/week)], and total energy intake. Medical conditions included a history of hypertension, diabetes, hypercholesterolemia, and cardiovascular diseases [CVD (heart attack, angina, and stroke)] for the general population, and additionally menopause status, hormone replacement therapy (HRT), oral contraceptive use, and hysterectomy for females.

### Statistical analyses

We conducted RRR to identify the obesity-related DP. The assumption that response variables (obesity-related indexes) need to be on the causal pathway between predictors (diets) and dependent variables (cancer) was verified by previous studies [11, 19, 20]. RRR procedure firstly used a linear combination of the pre-assigned Z-score of food groups to derive the factor loading of each food group and further to calculate obesity-related DP scores via the sum of food intakes weighted by corresponding unique value. Positive and negative factor loadings were related to increased and decreased obesity-related DP scores. Pearson correlation coefficients were estimated to reflect the correlation between obesity-related DP scores and response variables. The first DP was chosen from 2 derived-DPs, and it was used for all analyses since it explained the maximal variation. We selected the main food groups (|factor loading|> 0.2) to describe the first derived DP. The distributions of baseline characteristics of participants across quartiles of obesity-related DP Z-scores were displayed by frequency (relative ratio) for categorical variables or mean (standard deviation) for continuous variables. The differences in baseline characteristics across quartiles of obesity-related DP scores were examined by chi-squared or ANOVA test where appropriate. We assumed that the missing values are missing at random and used multiple imputation with 20 replications to impute these missing values for non-systematically missing covariates, based on a chained equation method and combined the results using Rubin’s rules. Details of missing proportions of covariates are listed in Additional file 1 (Table S3).

We assessed the associations of obesity-related DP with incident overall cancer and 19 cancer sites by conducting cox proportional hazards models weighted by the number of dietary assessments, with study time as the time-dependent variable. We assessed the relationships between obesity-related DP Z-scores (treated as quartiles and continuous variables, respectively) and the cancers by running cox models as follows: model 1 was unadjusted; model 2 was adjusted for age (continuous) and stratified by sex; model 3 was further stratified by study regions and adjusted for ethnicity, Townsend deprivation index, education level, smoking status, physical activity, and total energy intake per day (log-transformed); model 4 was further adjusted for hypertension, diabetes, hypercholesterolemia, and CVD, and additionally adjusted for HRT use, oral contraceptive use, and menopause status after excluding females with a history of hysterectomy (*n* = 2980) for cervix, ovary, and endometrium cancers. In model 4, HRT use and oral contraceptive use were additionally adjusted for premenopausal and postmenopausal breast cancer. A significant quadratic term for obesity-related DP Z-scores was added to the cox model when we found a lower Akaike information criterion (AIC) value for the model after including it. Moreover, a test for linear trends was also conducted by assigning the median values to each quartile of obesity-related DP Z-scores as a continuous variable. We calculated population-attributable fractions (PAFs) of the high quartile (combination of Q3 and Q4) of obesity-related DP Z-scores (exposure) for overall cancer at each follow-up year in model 3. The proportional hazards assumption was tested by Schoenfeld residuals (*P* > 0.05) and not violated for the main variables except for sex and study regions, which were included as stratified variables in cox models [21]. Besides, we explored the dose–response associations between obesity-related DP Z-scores and cancers by restricted cubic splines with knots determined by the minimum value of the AIC from 3 to 7 knots. In addition, subgroup analyses for each outcome across a combination of age groups and sex (male younger than 65, male aged 65 and older, female younger than 65, and female aged 65 and older) were conducted, with covariates in model 3. To quantify the mediation effect size of response variables (BMI and WHR), which were assumed on the pathway between obesity-related DP and cancers, and identify other potential mediators of interest based on prior knowledge [22, 23], we constructed a paralleled mediation model with adjustment for covariates in model 3 between obesity-related DP and overall cancer including response variables, systolic blood pressure, diastolic blood pressure, and log-transformed blood biomarkers (Glycated hemoglobin A1c, HbA1c; fasting blood glucose; triglycerides; Low-density lipoprotein, LDL; High-density lipoprotein, HDL; C-reactive protein) as mediators. We derived 95%CI of path-specific natural indirect effect and natural direct effect estimation based on 1,000 bootstrapped simulations. To clarify whether interested factors modified the associations between obesity-related DP and overall and site-specific cancers, we examined the heterogeneity using the likelihood ratio test comparing models with and without multiplicative terms (obesity-related DP Z-scores and possible moderators).

We implemented a series of sensitivity analyses to assess the robustness of our findings. We used average consumption of foods on at least 2 available assessments for analyses below unless otherwise specified. First, we re-ran cox models among individuals with complete data. Second, we re-ran cox models after excluding incident cancer cases within the first 2 years of follow-ups to minimize the potential impact of reverse causality. Third, we re-ran cox models for those who fully completed 5 dietary assessments. Fourth, to test these associations whether differ after considering the influence of competing risks of non-cancer death, we assessed the competing risk of non-cancer death on the associations using the sub-distribution method proposed by Fine and Grey [24]. Moreover, we set obesity indicators, which are frequently co-occurrence with other metabolic disorders [22], and other MetS components as response variables (BMI, waist-to-hip ratio, HDL, LDL, triglycerides, fasting blood glucose, HbA1c, systolic blood pressure, and diastolic blood pressure) to explore the associations between the MetS-related DP and cancers. To provide more insight into the temporal relationships between the obesity-related DP and cancer risk, we set BMI and WHR measured in 2014 as response variables and explore associations between the new obesity DP and cancers (Procedure for this section provided in Addition file 1). In addition, after using the same exclusion criteria in this study for the 84,163 individuals with 1 dietary questionnaire, we did another sensitive analysis among those with one or more dietary assessments by adding the remaining participants (*n *= 74,381) with 1 dietary questionnaire into the original study participants to verify the robustness of the main results, with the available average consumption of foods on one or more available assessments. All analyses above were adjusted for covariates in model 3 and weighted by the number of dietary assessments. Last, the RRR procedure was separately performed among individuals with 3 or more, 4 or more, or 5 dietary assessments using corresponding available average amounts of foods to examine the reliability of the obesity-related DP.

RRR procedure was performed by SAS (version 9.4; SAS Institute), and the remaining analyses were conducted by Stata (version 16; StataCorp LP) and R i386 4.1.2 (R Foundation for Statistical Computing); and two-sided *P*-values ≤ 0.05 were regarded as statistically significant. Due to the inflation of false-positive findings, the false-discovery rate-corrected *P* values were calculated by the Benjamini–Hochberg method and all *P*-values were corrected.

## Results

**Fig. 1:**
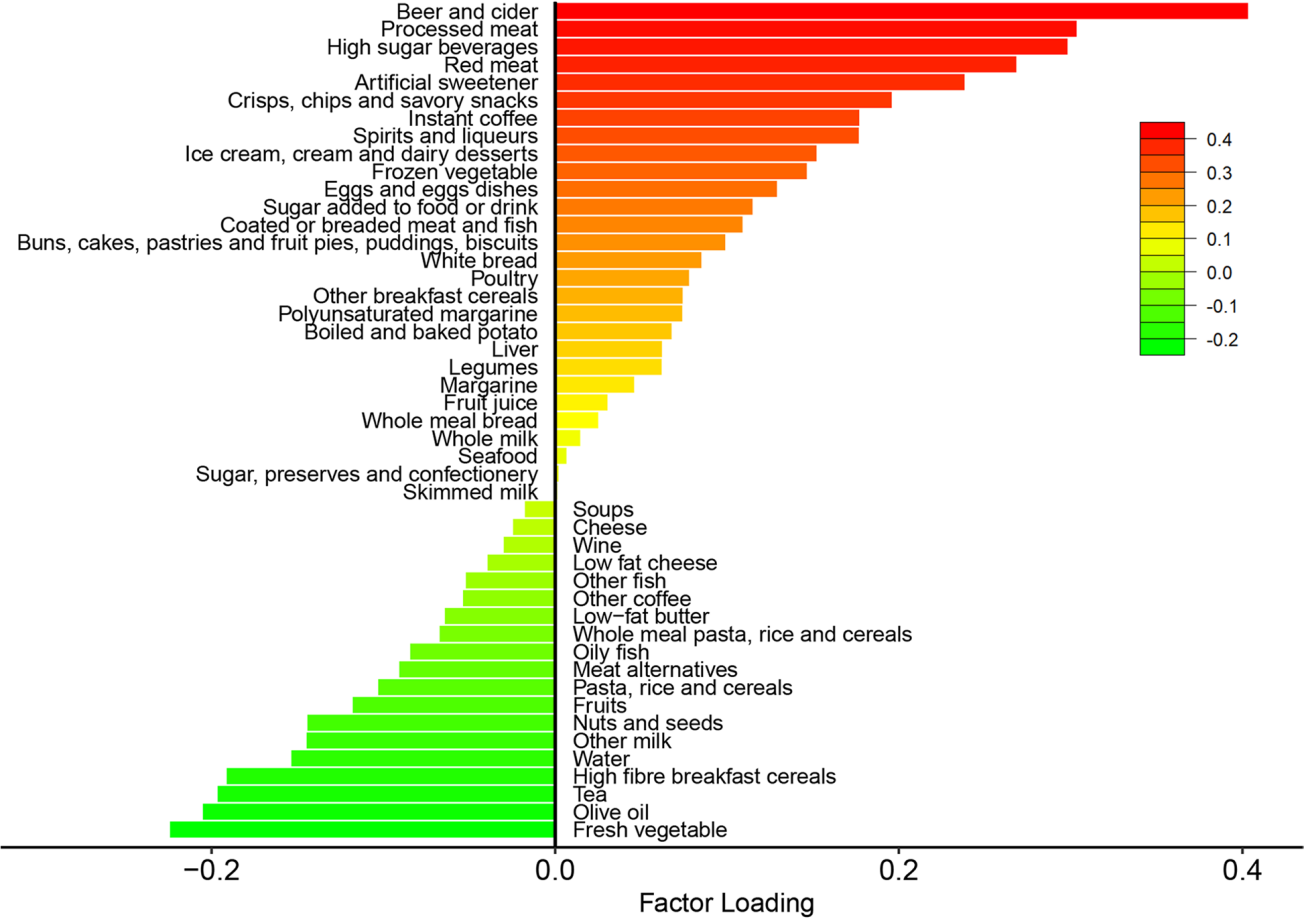
Factor loadings for obesity-related dietary pattern derived from reduced rank regression

The first DP explained the largest amount (11.2%) of variation in the response variables and showed positively strong correlations with WHR (*r* = 0.80) and BMI (*r* = 0.60) (Additional file 1: Table S4). As shown in Fig. 1, the obesity-related DP is characterized by a higher intake of beer and cider, processed meat, high sugar beverages, red meat, and artificial sweetener, and a lower intake of fresh vegetables, olive oil, tea, and high fiber breakfast cereals. The obesity-related DP Z-scores range from -3.97 to 7.27.

We included 114,289 participants, among whom 10,145 (8.9%) had incident cancers. The median follow-up period was 9.4 years for overall cancer. Baseline characteristics across quartiles of obesity-related DP Z-scores are displayed in Table 1. Individuals in the highest quartile tend to be male, less educated, current smokers, physical inactive, and higher in energy intake. Additionally, they tend to have worse health conditions, such as hypertension, diabetes, high cholesterol, and CVD at baseline (all *P*-values < 0.001).

**Table 1:** Baseline characteristics across quartiles of obesity-related DP Z-scores

Characteristics	Quartiles of the obesity-related dietary pattern				*P* value
	**Q1 (28,573)**	**Q2 (28,572)**	**Q3 (28,572)**	**Q4 (28,572)**	
**Dietary pattern Z-score**	-1.12 (0.37)	-0.40 (0.15)	0.18 (0.19)	1.34 (0.76)	
**Demographics**					
Men (%)	6869 (24.0)	10,217 (35.8)	14,251 (49.9)	20,108 (70.4)	< 0.001
Age (mean (SD))	55.71 (7.66)	56.09 (7.75)	56.05 (7.90)	55.48 (8.05)	< 0.001
White (%)	28,542 (99.9)	28,548 (100.0)	28,548 (100.0)	28,544 (99.9)	0.438
**Socioeconomic status**					
Townsend deprivation index (mean (SD))	-1.48 (2.91)	-1.74 (2.79)	-1.77 (2.76)	-1.53 (2.89)	< 0.001
Education (%)					
Higher or any school degree	26,696 (93.4)	25,725 (90.0)	25,112 (87.9)	23,890 (83.6)	< 0.001
Vocational qualifications	1064 (3.7)	1661 (5.8)	2007 (7.0)	2529 (8.9)	
Other	813 (2.8)	1186 (4.2)	1453 (5.1)	2153 (7.5)	
**Behavioral risk factors**					
Smoking status (%)					
Current	1277 (4.5)	1602 (5.6)	2056 (7.2)	3091 (10.8)	< 0.001
Never	17,765 (62.3)	17,486 (61.3)	16,485 (57.8)	13,875 (48.7)	
Previous	9478 (33.2)	9423 (33.1)	9969 (35.0)	11,539 (40.5)	
Physical activity (%)					
High	10,379 (42.1)	9225 (37.7)	9033 (36.8)	9189 (37.3)	< 0.001
Low	3489 (14.2)	4487 (18.4)	4883 (19.9)	5304 (21.5)	
Moderate	10,770 (43.7)	10,729 (43.9)	10,611 (43.3)	10,171 (41.2)	
**Energy intakes (kj/days)**	8023.28 (1903.00)	8358.48 (1915.20)	8876.94 (2006.16)	9882.35 (2290.08)	< 0.001
**Medical conditions**					
Hypertension (%)	4957 (17.4)	5816 (20.4)	6388 (22.4)	7706 (27.0)	< 0.001
Diabetes (%)	596 (2.1)	744 (2.6)	1096 (3.8)	1874 (6.6)	< 0.001
Hypercholesterolemia (%)	2602 (9.1)	3516 (12.3)	4397 (15.4)	6065 (21.2)	< 0.001
Cardiovascular diseases (%)	652 (2.3)	921 (3.2)	1189 (4.2)	1681 (5.9)	< 0.001
Oral contraceptive use (%)	18,521 (85.5)	15,724 (85.8)	12,270 (85.9)	7387 (87.4)	< 0.001
HRT use (%)	7100 (32.8)	6370 (34.8)	5172 (36.2)	3002 (35.6)	< 0.001
Menopause (%)	12,979 (59.8)	10,866 (59.2)	8086 (56.5)	4301 (50.8)	< 0.001
**Response variables**					
BMI (mean (SD))	25.07 (4.01)	26.22 (4.25)	27.11 (4.46)	28.47 (4.77)	< 0.001
WHR (mean (SD))	0.82 (0.08)	0.85 (0.08)	0.87 (0.08)	0.91 (0.08)	< 0.001

*BMI* Body mass index. *WHR* Waist-to-hip ratio

### Associations between dietary pattern and cancers

The PAFs of obesity-related DP decreased over the follow-up period, from 3.21% at 1 year to 3.01% at 10 years (Additional file 1**: **Fig. 1). The results for the associations of the DP with incident cancer are given in Table 2. In the crude cox model 1, higher adherence to obesity-related DP was linearly associated with increased risks of oral, esophagus, stomach, colorectal, liver, pancreas, lung, endometrium, bladder, and leukemia cancers; and the non-linear associations were observed for malignant melanoma, prostate, kidney, non-Hodgkin lymphoma, and multiple myeloma cancer, as well as overall cancer. In model 4 with multivariable adjustment, the nonlinear associations for overall and malignant melanoma cancer become positive linear associations (*P* < 0.05), in which HRs (95%CI) per 1-SD were 1.02 (1.01, 1.04) and 0.92 (0.87, 0.98). The linear positive associations for esophagus and bladder cancers become nonlinear associations, where the HRs (95%CI) per 1-SD for linear and quadric terms are 1.03 (0.94, 1.14) and 1.06 (1.02, 1.09) and 1.00 (0.93, 1.06) and 1.05 (1.03, 1.08). The monotonic linear associations for several cancer sites remain significant but slightly weaker, with HRs (95%CI) per 1-SD for oral 1.34 (1.12, 1.61), colorectal 1.08 (1.04, 1.12), lung 1.11 (1.06, 1.17), and endometrium 1.21 (1.11, 1.31). In model 4, compared with the lowest quartile of obesity-related DP Z-scores, individuals in the highest have a significantly increased cancer risk by 7% (overall cancer), 118% (oral), 20% (colorectal), 53% (liver), 35% (lung), 60% (endometrium), 65% (ovary), 43% (kidney), and 76% (thyroid). The remaining information can be found in Table 2.

**Table 2:** Associations of obesity-related DP Z-scores and quartiles with overall and 19 site-specific cancers in four separate models among general adults

Types of cancer	Linear term	Quadric term	Quartiles of dietary pattern Z-score				Corrected *P* for trend
			**Q1**	**Q2**	**Q3**	**Q4**	
Overall cancer							
Cases/Total number	10,145/114289		2244/28573	2448/28572	2631/28572	2822/28572	
Model 1	1.11 (1.09, 1.12) ***	0.98 (0.98, 0.99) ***	1 (Ref)	1.09 (1.06, 1.13) ***	1.19 (1.15, 1.23) ***	1.27 (1.23, 1.32) ***	< 0.001
Model 2	1.06 (1.05, 1.07) ***		1 (Ref)	1.04 (1.00, 1.07) *	1.10 (1.06, 1.14) ***	1.16 (1.12, 1.20) ***	< 0.001
Model 3	1.03 (1.02, 1.05) ***		1 (Ref)	1.04 (1.00, 1.07) *	1.10 (1.06, 1.14) ***	1.16 (1.12, 1.20) ***	< 0.001
Model 4	1.02 (1.01, 1.04) ***		1 (Ref)	1.02 (0.99, 1.05)	1.06 (1.02, 1.09) **	1.07 (1.03, 1.10) ***	0.001
Oral							
Cases/Total number	47/114289		7/28573	11/28572	11/28572	18/28572	
Model 1	1.38 (1.19, 1.61) ***		1 (Ref)	1.50 (0.85, 2.66)	1.52 (0.86, 2.68)	2.51 (1.49, 4.25) ***	0.001
Model 2	1.34 (1.13, 1.58) **		1 (Ref)	1.42 (0.80, 2.51)	1.36 (0.76, 2.43)	2.16 (1.24, 3.76) **	0.013
Model 3	1.33 (1.11, 1.59) **		1 (Ref)	1.42 (0.80, 2.51)	1.36 (0.76, 2.43)	2.16 (1.24, 3.76) **	0.025
Model 4	1.34 (1.12, 1.61) **		1 (Ref)	1.45 (0.81, 2.61)	1.41 (0.77, 2.56)	2.18 (1.21, 3.94) *	0.029
Esophagus							
Cases/Total number	222/114289		36/28573	58/28572	55/28572	73/28572	
Model 1	1.33 (1.25, 1.42) ***		1 (Ref)	1.46 (1.15, 1.86) **	1.49 (1.17, 1.89) **	1.96 (1.56, 2.46) ***	< 0.001
Model 2	1.12 (1.02, 1.22) *	1.05 (1.02, 1.09) **	1 (Ref)	1.26 (0.99, 1.61)	1.14 (0.89, 1.45)	1.37 (1.08, 1.74) **	0.045
Model 3	1.09 (0.99, 1.20)	1.05 (1.02, 1.09) **	1 (Ref)	1.26 (0.99, 1.61)	1.14 (0.89, 1.45)	1.37 (1.08, 1.74) **	0.284
Model 4	1.03 (0.94, 1.14)	1.06 (1.02, 1.09) **	1 (Ref)	1.20 (0.94, 1.53)	1.03 (0.80, 1.32)	1.10 (0.86, 1.42)	0.781
Stomach							
Cases/Total number	157/114289		20/28573	40/28572	42/28572	55/28572	
Model 1	1.30 (1.20, 1.41) ***		1 (Ref)	1.94 (1.43, 2.62) ***	1.96 (1.44, 2.65) ***	2.56 (1.91, 3.42) ***	< 0.001
Model 2	1.11 (1.02, 1.22) *		1 (Ref)	1.62 (1.19, 2.19) **	1.39 (1.02, 1.89) *	1.58 (1.16, 2.13) **	0.058
Model 3	1.04 (0.94, 1.15)		1 (Ref)	1.62 (1.19, 2.19) **	1.39 (1.02, 1.89) *	1.58 (1.16, 2.13) **	0.505
Model 4	1.02 (0.93, 1.13)		1 (Ref)	1.53 (1.13, 2.08) **	1.26 (0.92, 1.72)	1.27 (0.92, 1.74)	0.702
Colorectal							
Cases/Total number	1218/114289		239/28573	278/28572	320/28572	381/28572	
Model 1	1.19 (1.16, 1.23) ***		1 (Ref)	1.14 (1.03, 1.26) *	1.35 (1.22, 1.49) ***	1.61 (1.46, 1.77) ***	< 0.001
Model 2	1.14 (1.10, 1.18) ***		1 (Ref)	1.06 (0.96, 1.17)	1.19 (1.08, 1.32) ***	1.38 (1.25, 1.52) ***	< 0.001
Model 3	1.09 (1.05, 1.13) ***		1 (Ref)	1.06 (0.96, 1.17)	1.19 (1.08, 1.32) ***	1.38 (1.25, 1.52) ***	< 0.001
Model 4	1.08 (1.04, 1.12) ***		1 (Ref)	1.04 (0.94, 1.15)	1.12 (1.02, 1.24) *	1.20 (1.08, 1.33) ***	0.001
Liver							
Cases/Total number	145/114289		24/28573	40/28572	34/28572	47/28572	
Model 1	1.27 (1.17, 1.38) ***		1 (Ref)	1.74 (1.29, 2.34) ***	1.52 (1.12, 2.07) **	2.28 (1.71, 3.04) ***	< 0.001
Model 2	1.22 (1.11, 1.33) ***		1 (Ref)	1.61 (1.19, 2.17) **	1.33 (0.97, 1.81)	1.93 (1.43, 2.61) ***	0.001
Model 3	1.19 (1.07, 1.31) **		1 (Ref)	1.61 (1.19, 2.17) **	1.33 (0.97, 1.81)	1.93 (1.43, 2.61) ***	0.006
Model 4	1.11 (1.00, 1.23) *		1 (Ref)	1.51 (1.12, 2.05) **	1.19 (0.87, 1.63)	1.53 (1.12, 2.10) **	0.106
Pancreas							
Cases/Total number	287/114289		66/28573	65/28572	71/28572	85/28572	
Model 1	1.09 (1.02, 1.16) *		1 (Ref)	0.90 (0.74, 1.10)	1.07 (0.88, 1.30)	1.22 (1.01, 1.47) *	0.015
Model 2	1.07 (1.00, 1.15)		1 (Ref)	0.85 (0.70, 1.04)	0.99 (0.81, 1.20)	1.13 (0.93, 1.39)	0.126
Model 3	1.05 (0.97, 1.13)		1 (Ref)	0.85 (0.70, 1.04)	0.99 (0.81, 1.20)	1.13 (0.93, 1.39)	0.348
Model 4	1.02 (0.94, 1.10)		1 (Ref)	0.82 (0.67, 1.01)	0.93 (0.76, 1.13)	1.01 (0.81, 1.24)	0.702
Lung							
Cases/Total number	645/114289		130/28573	127/28572	158/28572	230/28572	
Model 1	1.24 (1.19, 1.29) ***		1 (Ref)	1.01 (0.88, 1.17)	1.27 (1.11, 1.45) ***	1.76 (1.55, 2.00) ***	< 0.001
Model 2	1.29 (1.23, 1.35) ***		1 (Ref)	0.99 (0.86, 1.14)	1.25 (1.09, 1.44) **	1.87 (1.63, 2.14) ***	< 0.001
Model 3	1.13 (1.08, 1.19) ***		1 (Ref)	0.99 (0.86, 1.14)	1.25 (1.09, 1.44) **	1.87 (1.63, 2.14) ***	< 0.001
Model 4	1.11 (1.06, 1.17) ***		1 (Ref)	0.94 (0.82, 1.09)	1.10 (0.96, 1.27)	1.35 (1.17, 1.56) ***	< 0.001
Malignant melanoma							
Cases/Total number	527/114289		124/28573	126/28572	152/28572	125/28572	
Model 1	0.98 (0.93, 1.04)	0.96 (0.93, 1.00) *	1 (Ref)	0.97 (0.84, 1.12)	1.20 (1.04, 1.37) *	0.93 (0.80, 1.08)	0.748
Model 2	0.94 (0.89, 0.99) *		1 (Ref)	0.94 (0.81, 1.08)	1.13 (0.98, 1.30)	0.88 (0.75, 1.03)	0.367
Model 3	0.93 (0.88, 0.99) *		1 (Ref)	0.94 (0.81, 1.08)	1.13 (0.98, 1.30)	0.88 (0.75, 1.03)	0.348
Model 4	0.92 (0.87, 0.98) **		1 (Ref)	0.92 (0.80, 1.06)	1.10 (0.96, 1.27)	0.86 (0.73, 1.01)	0.373
Premenopausal breast							
Cases/Total number	809/26612		262/8725	218/7489	212/6235	117/4163	
Model 1	0.99 (0.94, 1.04)		1 (Ref)	0.92 (0.83, 1.02)	1.10 (0.99, 1.23)	0.89 (0.78, 1.01)	0.587
Model 2	0.99 (0.95, 1.04)		1 (Ref)	0.92 (0.83, 1.02)	1.10 (0.99, 1.22)	0.89 (0.78, 1.02)	0.620
Model 3	0.97 (0.92, 1.02)		1 (Ref)	0.92 (0.83, 1.02)	1.10 (0.99, 1.22)	0.89 (0.78, 1.02)	0.284
Model 4	0.97 (0.92, 1.02)		1 (Ref)	0.90 (0.81, 1.00)	1.06 (0.95, 1.18)	0.84 (0.73, 0.96) **	0.237
Postmenopausal breast							
Cases/Total number	1190/36254		401/12986	388/10871	246/8091	155/4306	
Model 1	1.04 (0.99, 1.08)		1 (Ref)	1.15 (1.06, 1.24) ***	0.98 (0.89, 1.07)	1.16 (1.04, 1.29) **	0.041
Model 2	1.03 (0.99, 1.08)		1 (Ref)	1.14 (1.05, 1.24) **	0.97 (0.89, 1.07)	1.16 (1.04, 1.30) **	< 0.001
Model 3	1.00 (0.96, 1.05)		1 (Ref)	1.14 (1.05, 1.24) **	0.97 (0.89, 1.07)	1.16 (1.04, 1.30) **	0.003
Model 4	0.99 (0.95, 1.04)		1 (Ref)	1.11 (1.02, 1.20) *	0.92 (0.83, 1.01)	1.05 (0.93, 1.17)	0.009
Cervix							
Cases/Total number	36/62844		12/21704	8/18355	12/14321	4/8464	
Model 1	1.13 (0.88, 1.44)		1 (Ref)	0.86 (0.49, 1.51)	1.67 (1.01, 2.75) *	0.77 (0.36, 1.66)	0.148
Model 2	1.14 (0.89, 1.46)		1 (Ref)	0.86 (0.49, 1.51)	1.67 (1.01, 2.76) *	0.78 (0.36, 1.69)	0.811
Model 3	1.16 (0.90, 1.51)		1 (Ref)	0.86 (0.49, 1.51)	1.67 (1.01, 2.76) *	0.78 (0.36, 1.69)	0.661
Model 4	1.15 (0.87, 1.53)		1 (Ref)	0.89 (0.47, 1.67)	1.75 (0.98, 3.11)	0.78 (0.32, 1.91)	0.477
Endometrium							
Cases/Total number	288/62844		86/21704	85/18355	72/14321	45/8464	
Model 1	1.14 (1.05, 1.24) **		1 (Ref)	1.23 (1.03, 1.46) *	1.28 (1.07, 1.54) **	1.41 (1.14, 1.74) **	0.088
Model 2	1.17 (1.08, 1.27) ***		1 (Ref)	1.22 (1.02, 1.45) *	1.28 (1.07, 1.54) **	1.49 (1.21, 1.84) ***	0.514
Model 3	1.21 (1.11, 1.32) ***		1 (Ref)	1.22 (1.02, 1.45) *	1.28 (1.07, 1.54) **	1.49 (1.21, 1.84) ***	0.638
Model 4	1.21 (1.11, 1.31) ***		1 (Ref)	1.26 (1.06, 1.50) **	1.38 (1.14, 1.66) ***	1.60 (1.28, 1.99) ***	0.563
Ovary							
Cases/Total number	208/62844		58/21704	69/18355	44/14321	37/8464	
Model 1	1.09 (0.99, 1.20)		1 (Ref)	1.52 (1.24, 1.86) ***	1.23 (0.98, 1.55)	1.64 (1.27, 2.10) ***	< 0.001
Model 2	1.10 (1.00, 1.22)		1 (Ref)	1.51 (1.23, 1.85) ***	1.24 (0.98, 1.56)	1.70 (1.32, 2.19) ***	0.367
Model 3	1.07 (0.96, 1.18)		1 (Ref)	1.51 (1.23, 1.85) ***	1.24 (0.98, 1.56)	1.70 (1.32, 2.19) ***	0.342
Model 4	1.08 (0.97, 1.20)		1 (Ref)	1.46 (1.19, 1.80) ***	1.22 (0.97, 1.54)	1.65 (1.27, 2.14) ***	0.563
Prostate							
Cases/Total number	2209/51445		310/6869	441/10217	650/14251	808/20108	
Model 1	0.99 (0.96, 1.02)	0.97 (0.95, 0.98) ***	1 (Ref)	0.98 (0.90, 1.06)	1.04 (0.96, 1.12)	0.90 (0.83, 0.97) **	0.113
Model 2	0.98 (0.96, 1.00)		1 (Ref)	0.95 (0.87, 1.03)	1.03 (0.95, 1.11)	0.94 (0.87, 1.02)	0.138
Model 3	0.97 (0.95, 1.00) *		1 (Ref)	0.95 (0.87, 1.03)	1.03 (0.95, 1.11)	0.94 (0.87, 1.02)	0.879
Model 4	0.98 (0.96, 1.01)		1 (Ref)	0.95 (0.87, 1.03)	1.03 (0.95, 1.11)	0.95 (0.87, 1.03)	0.781
Kidney							
Cases/Total number	295/114289		44/28573	66/28572	83/28572	102/28572	
Model 1	1.36 (1.26, 1.48) ***	0.95 (0.92, 0.99) *	1 (Ref)	1.42 (1.14, 1.76) **	1.88 (1.53, 2.31) ***	2.28 (1.86, 2.78) ***	0.714
Model 2	1.16 (1.08, 1.23) ***		1 (Ref)	1.26 (1.01, 1.57) *	1.52 (1.23, 1.87) ***	1.69 (1.37, 2.09) ***	0.678
Model 3	1.11 (1.04, 1.20) **		1 (Ref)	1.26 (1.01, 1.57) *	1.52 (1.23, 1.87) ***	1.69 (1.37, 2.09) ***	0.638
Model 4	1.08 (1.00, 1.16) *		1 (Ref)	1.20 (0.97, 1.50)	1.40 (1.13, 1.73) **	1.43 (1.14, 1.78) **	0.702
Bladder							
Cases/Total number	492/114289		89/28573	99/28572	130/28572	174/28572	
Model 1	1.33 (1.27, 1.39) ***		1 (Ref)	1.07 (0.91, 1.26)	1.40 (1.20, 1.64) ***	1.97 (1.70, 2.28) ***	0.001
Model 2	1.07 (1.00, 1.14)	1.05 (1.03, 1.07) ***	1 (Ref)	0.88 (0.75, 1.04)	0.99 (0.85, 1.16)	1.22 (1.05, 1.43) *	< 0.001
Model 3	1.02 (0.96, 1.09)	1.05 (1.03, 1.08) ***	1 (Ref)	0.88 (0.75, 1.04)	0.99 (0.85, 1.16)	1.22 (1.05, 1.43) *	< 0.001
Model 4	1.00 (0.93, 1.06)	1.05 (1.03, 1.08) ***	1 (Ref)	0.85 (0.72, 1.00)	0.91 (0.78, 1.07)	1.01 (0.86, 1.19)	< 0.001
Thyroid							
Cases/Total number	69/114289		17/28573	13/28572	17/28572	22/28572	
Model 1	1.11 (0.98, 1.27)		1 (Ref)	0.91 (0.60, 1.39)	1.26 (0.86, 1.86)	1.41 (0.96, 2.06)	0.001
Model 2	1.30 (1.13, 1.50) ***		1 (Ref)	0.99 (0.65, 1.50)	1.54 (1.04, 2.28) *	2.10 (1.41, 3.14) ***	0.001
Model 3	1.26 (1.09, 1.47) **		1 (Ref)	0.99 (0.65, 1.50)	1.54 (1.04, 2.28) *	2.10 (1.41, 3.14) ***	0.007
Model 4	1.22 (1.05, 1.43) *		1 (Ref)	0.92 (0.60, 1.41)	1.38 (0.92, 2.06)	1.76 (1.15, 2.69) *	0.005
Non-Hodgkin lymphoma							
Cases/Total number	447/114289		93/28573	120/28572	122/28572	112/28572	
Model 1	1.10 (1.03, 1.17) **	0.95 (0.91, 0.98) **	1 (Ref)	1.25 (1.07, 1.46) **	1.23 (1.05, 1.43) **	1.17 (1.00, 1.37)	0.001
Model 2	1.00 (0.95, 1.06)		1 (Ref)	1.17 (1.00, 1.37) *	1.10 (0.94, 1.29)	1.02 (0.86, 1.21)	0.221
Model 3	0.99 (0.93, 1.06)		1 (Ref)	1.17 (1.00, 1.37) *	1.10 (0.94, 1.29)	1.02 (0.86, 1.21)	0.206
Model 4	0.98 (0.92, 1.04)		1 (Ref)	1.15 (0.99, 1.35)	1.06 (0.91, 1.25)	0.95 (0.80, 1.13)	0.477
Multiple myeloma							
Cases/Total number	227/114289		50/28573	51/28572	68/28572	58/28572	
Model 1	1.17 (1.06, 1.29) **	0.87 (0.81, 0.93) ***	1 (Ref)	1.04 (0.82, 1.31)	1.38 (1.11, 1.72) **	1.17 (0.93, 1.47)	< 0.001
Model 2	1.11 (1.00, 1.23) *	0.89 (0.83, 0.95) ***	1 (Ref)	0.97 (0.77, 1.23)	1.25 (1.00, 1.56)	1.05 (0.82, 1.33)	< 0.001
Model 3	1.09 (0.98, 1.22)	0.89 (0.83, 0.95) ***	1 (Ref)	0.97 (0.77, 1.23)	1.25 (1.00, 1.56)	1.05 (0.82, 1.33)	< 0.001
Model 4	1.11 (0.99, 1.23)	0.89 (0.83, 0.95) ***	1 (Ref)	0.97 (0.77, 1.23)	1.24 (0.99, 1.56)	1.05 (0.82, 1.35)	0.007
Leukemia							
Cases/Total number	303/114289		65/28573	70/28572	78/28572	90/28572	
Model 1	1.12 (1.05, 1.19) ***		1 (Ref)	1.13 (0.93, 1.37)	1.26 (1.04, 1.52) *	1.46 (1.21, 1.75) ***	< 0.001
Model 2	1.02 (0.95, 1.09)		1 (Ref)	1.00 (0.82, 1.22)	1.01 (0.83, 1.23)	1.10 (0.90, 1.34)	0.001
Model 3	1.02 (0.95, 1.10)		1 (Ref)	1.00 (0.82, 1.22)	1.01 (0.83, 1.23)	1.10 (0.90, 1.34)	0.144
Model 4	1.01 (0.94, 1.09)		1 (Ref)	1.00 (0.82, 1.22)	1.01 (0.83, 1.23)	1.08 (0.88, 1.33)	0.477

Model 1 was unadjusted; model 2 was adjusted for age (continuous) and stratified by sex; model 3 was further stratified by study regions and adjusted for ethnicity, Townsend deprivation index, education level, smoking status, physical activity, and total energy intake per day (log-transformed); model 4 was further adjusted for hypertension, diabetes, hypercholesterolemia, and CVD, and additionally adjusted for HRT use, oral contraceptive use, and menopause status after excluding females with a history of hysterectomy (*n* = 2980) for cervix, ovary, and endometrium cancers. In model 4, HRT use and oral contraceptive use were additionally adjusted for premenopausal and postmenopausal breast cancer. The hazard ratio for linear and quadric term denoted that an increase of 1-SD in dietary pattern score.* P* for trend tests were conducted by including the median score of each pattern quartile as a continuous variable in the models and corrected due to the inflation of false discovery rate. *Crude *P* values < 0.05; **crude *P* values < 0.01, ***crude *P* values < 0.001. HR (95%CI) in bold represents false discovery rate-corrected *P* values < 0.05

### Dose–response associations between obesity-related DP and cancers

**Fig. 2:**
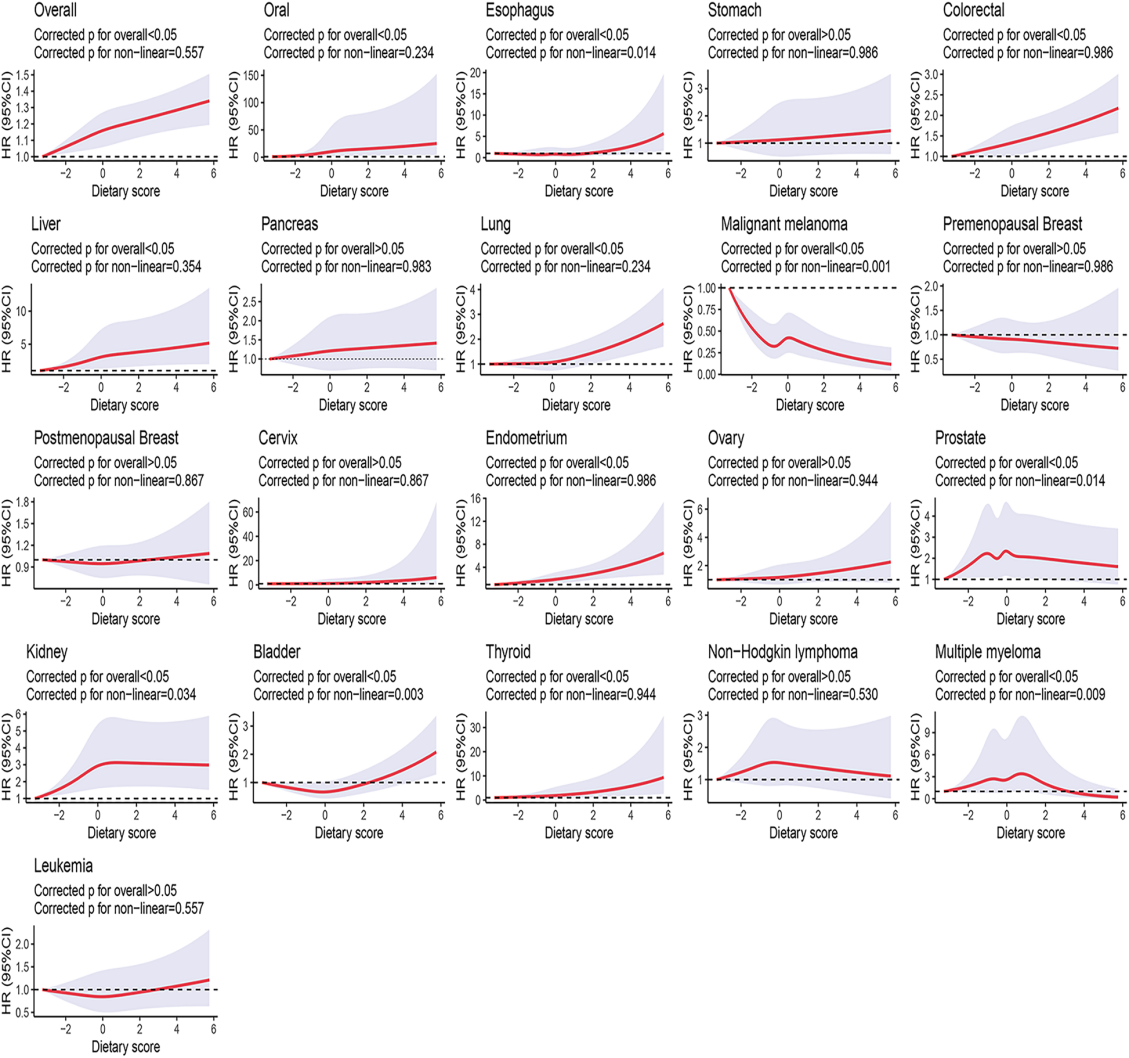
Dose–response associations between obesity-related DP Z-scores and risks of overall cancer and 19 site-specific cancers. All analyses were stratified by sex and study region and adjusted for age, ethnicity, Townsend deprivation index, education attainment, physical activity, smoking status, total energy intake per day (log-transformed), and history of CVD. Models for cervix, ovary, and endometrium cancers were adjusted for HRT use, oral contraceptive use, and menopause after excluding females with a history of hysterectomy (*n* = 2980). Additionally, Model for premenopausal and postmenopausal cancer was adjusted for HRT use and oral contraceptive use. We set the lowest dietary Z-score as reference

After exploring the dose–response associations between obesity-related DP Z-scores and cancers based on model 3, we further confirmed that there were meaningful nonlinear associations of obesity-related DP Z-scores with cancers of the esophagus (J-shaped, the inflection point at around a Z-score of 2 [the highest quartiles of obesity-related DP Z-scores]), malignant melanoma (fast-to-low decreased), prostate (inverse U-shape, the highest HR at around a Z-score of 0), kidney (inverse L-shape, the inflection point at around a Z-score of 0 [the third quartiles of obesity-related DP Z-scores]), bladder (U-shape, the lowest HR at around a Z-score of 0), and multiple myeloma (near inverse U-shape, the highest HR at around a Z-score of 1 [the highest quartiles of obesity-related DP Z-scores]) (all corrected *P* for nonlinear < 0.05, Fig. 2).

### Associations between obesity-related DP and cancers across combinations of age groups and sexes

**Fig. 3:**
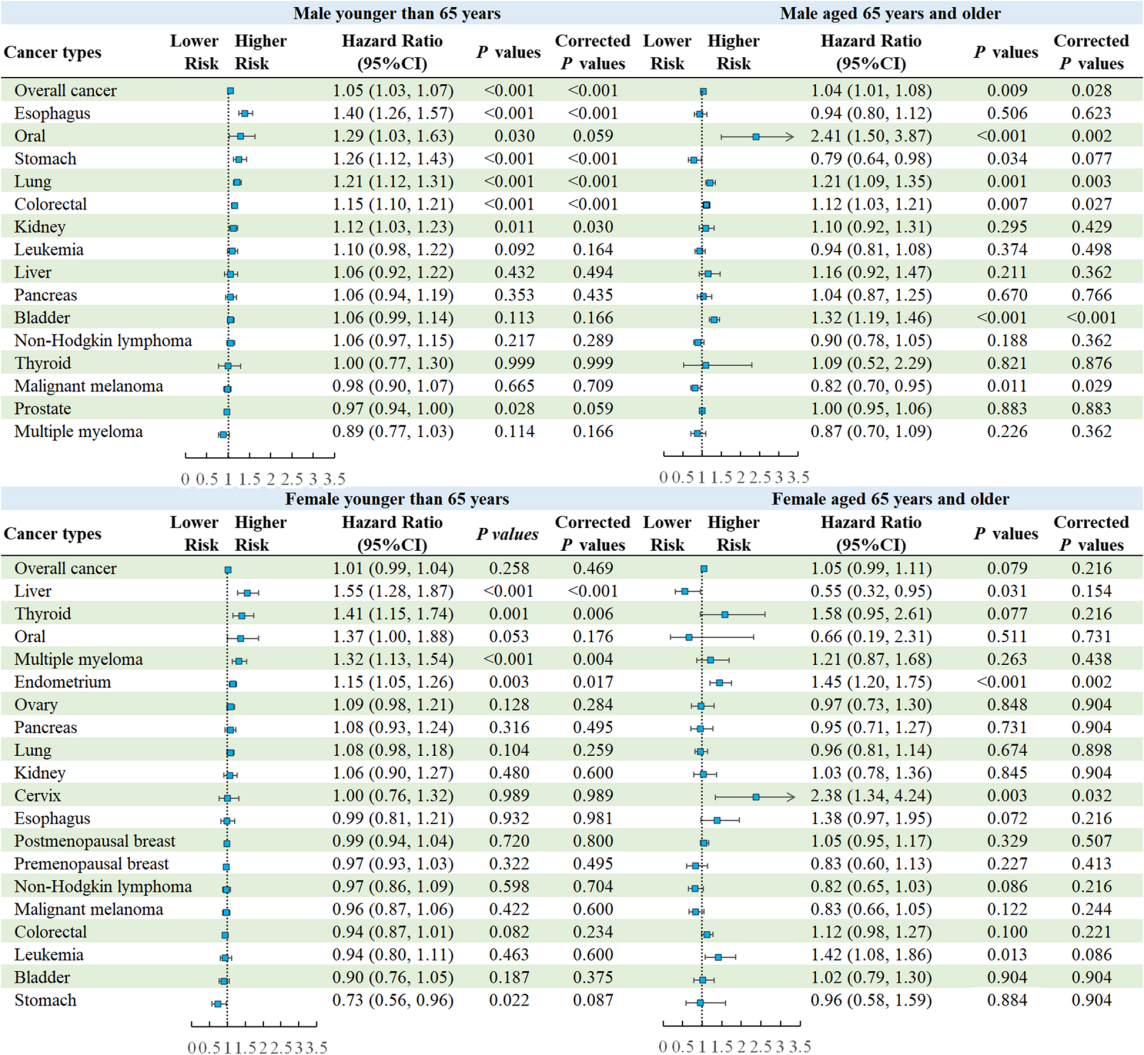
Associations between obesity-related DP Z-scores and risk of overall cancer by a combination of age groups and sexes. All analyses were stratified by sex and study region and adjusted for age, ethnicity, Townsend deprivation index, education attainment, physical activity, smoking status, and total energy intake per day (log-transformed)

We found that changes in HRs between the crude model 1 and age- and sex-adjusted model 2 were pronounced for almost all cancer types and that there was significant multiplicative interaction between age group (< 65 and ≥ 65 years) and obesity-related DP (*P*-interaction for age group < 0.001) for overall cancer. Hence, we further explored these associations in four subpopulations grouped in terms of a combination of age groups and sexes. In analyses for males younger than 65, we found that higher obesity-related DP Z-scores were associated with an increased risk of overall cancer (adjusted-HRs (95% CI) increase per 1-SD: 1.05, 1.03–1.07) and five site-specific cancers (esophagus, stomach, lung, colorectal, and kidney), whereas they were associated with an elevated risk of overall cancer (adjusted-HRs (95% CI) increase per 1-SD: 1.04, 1.01–1.08) and four site-specific cancers (oral, lung, colorectal, and bladder) and a reduced risk of malignant melanoma among males aged 65 and over. In analyses for females younger than 65, higher obesity-related DP Z-scores were associated with an increased risk of four site-specific cancers (liver, thyroid, multiple myeloma, and endometrium). However, higher obesity-related DP Z-scores were associated with an increased risk of two site-specific cancers (endometrium and cervix) among females aged 65 and over (Fig. 3).

### Parallel mediation model

**Fig. 4:**
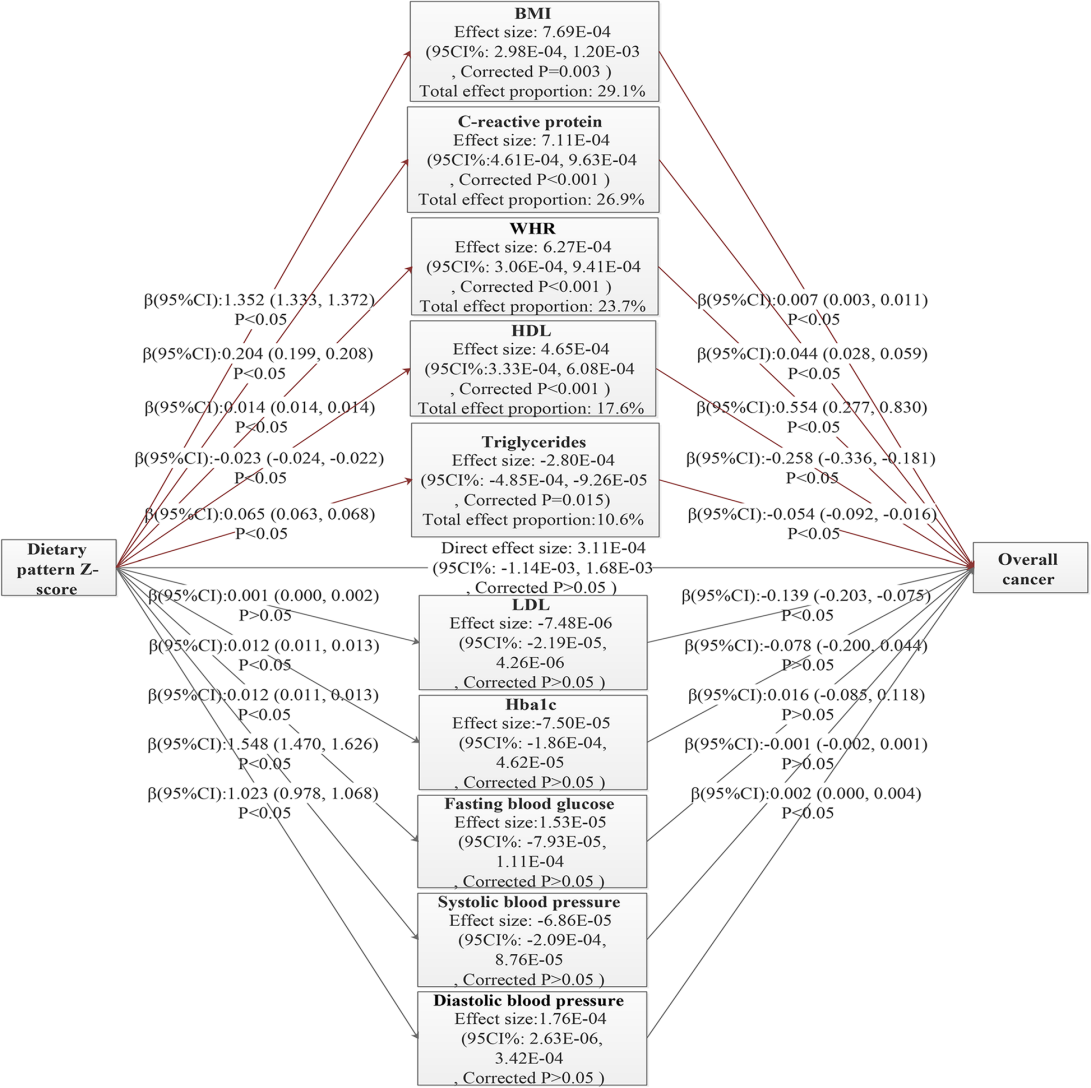
Conceptual diagram for paralleled mediation model. All analyses were adjusted for age, sex, ethnicity, study region, Townsend deprivation index, education attainment, physical activity, smoking status, and total energy intake per day (log-transformed)

The adjusted parallel mediation model indicated that the association between the obesity-related DP and overall cancer was fully mediated by five inter-mediators (BMI, WHR, HDL, C-reactive protein, and triglycerides). The total effect proportions of them were 29.1% (BMI), 26.9% (C-reactive protein), 23.7% (WHR), 17.6% (HDL), and 10.6% (triglycerides). As we expected, the relationship was mainly mediated by the response variables, and the direct effect was not statistically significant (*P* > 0.05). (Seeing the parameters in detail in Fig. 4).

### Sensitivity analyses

In sensitivity analysis, the associations between obesity-related DP Z-scores and each outcome remained robust in the context of complete data analysis and excluding the first 2-year follow-up. However, when analyses were restricted to those with five dietary assessments, we found that these associations were stronger. The competing risk models for each outcome suggested that the main results are robust (Additional file 1**: **Table S5). The heterogeneity analyses for overall cancer suggested that the association is modified by age, and that the relationship between the obesity-related DP and overall cancer tend to be less pronounced in adults younger than 65 years (Additional file 1**: **Table S7, *P*-interaction < 0.001). The remaining results of heterogeneity in the associations between the obesity-related DP and site-specific cancer sites are presented in (Additional file 1) Table S6-S12**.** The obesity-related DP and the main associations are nearly robust as those using MetS-related components (Additional file 1: Table S13, Fig S2) or obesity-related indicators measured at an intermediate time point (Additional file 1**: **Table S13, Fig. S3) as response variables. Our main findings were robust to the results after additionally including those with one dietary assessment (Additional file 1**: **Table S14, Fig. S4). Moreover, the obesity-related DPs from RRR analyses are similar regardless of the number of 24-h dietary assessments. (Additional file 1: Fig. S5-S7).

## Discussion

In this prospective study, we developed an obesity-related DP, which was characterized by a higher intake of beer and cider, processed meat, high sugar beverages, red meat, and artificial sweetener, and a lower intake of fresh vegetables, olive oil, tea, and high fiber breakfast cereals. General individuals in the highest versus the lowest obesity-related DP quartile were at elevated risk of overall cancer and oral, colorectal, liver, lung, endometrial, ovarian, kidney, and thyroid cancers. Moreover, the obesity-related DP was associated with different cancer sites among four subpopulations grouped by a combination of age groups and sexes. Additionally, we validated that the association between obesity-related DP and overall cancer was mainly mediated by the BMI and WHR (two response variables) and along with C-reactive protein and HDL and triglycerides. These findings have meaningful clinical and public health implications in the prevention of cancers through dietary approaches among the UK population.

From the multi-pathways between DPs and cancer, we chose one of the most effective pathways by setting the proxy indicators of obesity as the inter-mediator to bridge the indirect relationship between the obesity-related DP and cancer because obesity, an important established risk factor of several site-specific cancers [12], is the second biggest risk factor of cancers in the UK [25], following smoking. Although the variations of nutrients as more proximal response variables versus biomarkers can be substantially explained, it may violate the independence assumption for response variables when using predictors (food groups) and response variables from dietary assessment tools simultaneously [26]. Compared to 11.2% variation in response variables of our study, we noticed that previous studies also displayed low variations ranging from 1.7% to 4.2% [27–29] using biomarkers as response variables for RRR. Previous studies showed consistent results on certain cancer sites. For example, a meta-analysis of 93 studies suggested that prudent or healthy DPs are associated with a decreased risk of breast, colorectal, and lung cancer [30]. Additionally, the Western diet has been associated with a higher risk of colorectal [31, 32], breast [33, 34], esophageal [35], pancreatic [36], ovarian [37], and prostate cancers [38]. Likewise, we found that obesity-related DP was associated with overall and site-specific cancers in the digestive system. Moreover, we detected that certain non-digestive cancer sites, which have been less reported on, such as lung, kidney, bladder, endometrium, ovary, malignant melanoma, and multiple myeloma, were linked to the obesity-related DP. In total, we provided clues about potential relationships between the obesity-related DP and various cancer sites based on the given pathways.

Our study also systematically assessed the role of the obesity-related DP in the progress of a wide range of cancer sites. In the present study, beer and cider were in the unhealthy food group with the highest factor loading. A similar analysis between drink types and diseases also showed that beer and cider consumption was associated with a 14% higher risk of cancer and increased risks of CVD and all-cause mortality in the general UK population after excluding non-drinkers [39]. Red and processed meats, important components of the obesity-related DP and the Western diet, also play nonnegligible roles in carcinogenesis. An umbrella review summarized 72 meta-analyses and established that red and processed meat were separately associated with nine and 10 types of cancer, respectively, which was the same as our findings at specific cancer sites (colorectal, endometrium, esophagus, and lung) [40]. With urbanization and economic growth, sugar-sweetened and artificially sweetened beverages, major sources of added sugars in the diet, have become more popular in many populations, but they cause weight gain and an increased risk of cancer and other chronic diseases. Moreover, the adverse effects of sugar-based beverages on health outcomes, which have been shown by numerous cohort studies and clinical trials, were also demonstrated by Malik et al. [41]. Moreover, a meta-analysis selected 27 of 64 studies and found that sugar-sweetened beverages were positively associated with breast and prostate cancer risk and tended to be associated with colorectal and pancreatic cancer risk [42], which is partly in line with the results of our study. As to health factors in the obesity-related DP in this study, fresh vegetables and olive oil were major contributors to higher negative factor loadings, which were related to a lower extent of obesity. The Mediterranean diet also recommends higher intakes of fresh vegetables and olive oil, which similar studies have established to be related to a lower risk of colorectal cancer (RR _observational_: 0.82, 95% CI: 0.75 to 0.88; *n* = 11 studies) [43]. Further, a higher intake of vegetables has been found to decrease the risk of lung cancer by 8% (*n* = 25 studies) [44] and overall cancer by 10% (*n* = 15 studies) [45]. From a biochemical perspective, the chemopreventive activity of phenolic compounds in olive oil demonstrated an ability to inhibit oxidative DNA damage in several human and animal models [46], and other evidence also suggests the favorable effects of phenolic compounds on free radicals, inflammation, and carcinogenesis [47, 48]. Moreover, the beneficial effects of fiber and tea, both high in negative factor loading on cancers, have also been observed. Dietary fiber improves glucose and lipid parameters via the short-chain fatty acids produced by microbial fermentation [49] and inhibits the carcinogenic effects of N-nitroso compounds by acting as a nitrite scavenger [50]. Tea is one of the most popular beverages worldwide, and the protective effect of tea on six cancer sites has also been observed [51]. Tea is rich in various polyphenols, which may inhibit tumor formation and growth through its antioxidative and potentially antiproliferative roles [52]. Moreover, the consumption of alcohol in beer and cider generates the ethanol's metabolite acetaldehyde in blood circulation causing DNA damage and block DNA synthesis and repair [53]. Meat is also rich in harmful components such as heterocyclic amines, polycyclic aromatic hydrocarbons, and nitrate, which can impact the development of cancer [40]. Taken together, the compositions and contributions of the obesity-related DP have demonstrated a reasonable impact on the onset of cancers, but previous studies have shown borderline associations. In contrast, we developed an obesity-related DP, which was more strongly and widely associated with overall cancer and multiple site-specific cancers.

We observed different nonlinear association patterns for site-specific cancers; the inflection points were consistently at around 0 or 2 in terms of obesity-related DP Z-scores. The “platform-to-increase” risk for esophageal cancer might be explained by the previous evidence that suggests the opposite effects of BMI on the esophagus cancer type (adenocarcinoma versus squamous cell carcinoma) [54], whose constituent ratio might result in the J-shaped association. The “fast-to-low decrease” relationship for malignant melanoma cancer might reflect a real nonlinear biological association or partly be attributed to the disproportional contributions of the obesity-related DP to body fat-free mass and fat mass, whose opposite effects (i.e., adverse vs. protective) on malignant melanoma cancer were suggested by a study in UKB [55]. The inverse U-shaped relationship for prostate cancer might be caused by delayed or missed diagnoses in people with a high BMI, which is supported by the finding of the opposite effects of BMI on localized and advanced prostate cancer [56]. Moreover, the slight protective effect of the obesity-related DP on bladder cancer at the lower scale of the obesity-related DP Z-score could be attributed to heterogeneity across smoking status because the opposite effects were found among nonsmokers (protective) and current/previous (adverse) smokers (Additional file 1: Table S8, *P* for interaction < 0.001). Similarly, heterogeneity across sex for multiple myeloma cancer in the opposite effects of DP Z-score might explain the inverse U-shaped association. These heterogeneities indicate that there are different mechanisms or combinations of mechanisms associated with different cancer sites among different subgroups. Moreover, another observational study in UKB suggested different patterns of nonlinear associations between C-reactive protein (potential mediator) and cancer sites such as the kidney [23]. Taken together, the pooled effect of mediators might partly explain these complex nonlinear relationships, but the underlying mechanisms must be explored further.

Obesity is not merely an excess of adipose tissue; it also accompanies metabolic dysfunction (hyperglycemia, hypertension, and dyslipidemia) and local adipose tissue inflammation [57]. Indeed, the unfavorable effects of obesity on cancer development and progression are attributable to disruptions in adipokines, sex hormones, inflammation, and insulin metabolism [58]. Likewise, we found that the obesity-related DP could play a role in related inflammation pathways because C-reactive protein, one of the proxy indicators of inflammation, was the potential mediator in this study. This indicates that the obesity-related DP induces a local or systematic inflammation environment in the body, along with excess and abnormal fat accumulations. Moreover, HDL, as another mediator, partly bridged the connection between the obesity-related DP and overall cancer. As we know, HDL plays a key role in regulating cholesterol as well as anti-inflammation, antioxidation, and immune modulation, which is important in cancer incidence. Additionally, there is an assumption that HDL may facilitate the exchange of cholesterol between healthy and cancer cells [59].

In the present study, we used two or more dietary assessments to reflect standard intakes. Then, we constructed all of the models weighted by the number of dietary assessments to make our results reliable. Moreover, we set the last dietary assessment date as baseline time rather than the date of recruitment or first dietary assessment to avoid reverse causality. However, the insufficient number of cases in some cancer sites (oral, cervix, and thyroid) may have led to statistical inefficiency owing to the strict inclusion criteria in this study. Another limitation of our study was that dietary intakes were measured by multiple 24-h online dietary assessments, which were dependent on self-reporting, which may have caused misreporting and recall bias. To reflect the standard intake and capture reliable estimates, we used dietary data from a minimum of two 24-h online dietary assessments to derive the obesity-related DP, and then we compared it with the DP derived from distinct times dietary questionnaires to validate the stability of the obesity-related DP. Moreover, the response variables and dietary intakes might change over time. However, our study did not reflect long-term changes in dietary intake and body weight. However, we used response variables measured at an intermediate time point to validate the robustness of the results. Because this study was observational and confounded by potential unadjusted confounding factors, it is premature to infer causality. Finally, our posterior data-driven approach and regional dietary cultures make it difficult to generalize our results to other populations.

## Conclusions

Overall, we found that the obesity-related DP was strongly associated with a higher risk of overall cancer and multiple site-specific cancers. In general, in UK adults, adherence to the obesity-related DP was linearly associated with an increased risk of overall cancer and three site-specific cancers (oral, colorectal, and liver) in the digestive system and three site-specific cancers outside of the digestive system, and it was nonlinearly associated with six site-specific cancer sites. In the future, further studies with longer follow-up times and larger sample sizes will be conducted to identify other similar DPs.

## Supplementary Information

**Additional file 1: Table S1.** The contents of food groups.** TableS2. **ICD-10 code of 19 types of cancer.** Table S3. **Missing values and proportionsof covariates.** Table S4. **Explained variationin response variables for eachobesity-related dietary pattern as assessed using reduced rank regression andcorrelation coefficient between obesity-related dietary patterns and responsevariables.** Table S5. **Multivariable HRs and 95% CIs of the effect of dietarypattern on total and 19 site-specific cancers from, complete case analysis,excluding first 2 years incident cancer case, only including participants with5 times dietary assessments, and considering competing risk of non-cancerdeath. **Table S6. **Association between dietary pattern overall and site-specificcancers by sex.** Table S7.** Association between dietary pattern overall and site-specificcancers by age group.** Table S8. **Association between dietary pattern overall andsite-specific cancers by smoking status.** Table S9. **Association between dietarypattern overall and site-specific cancers by physical activity.** Table S10. **Association between dietary pattern overall and site-specific cancers bydiabetes.** Table S11. **Association between dietary pattern overall and site-specificcancers by hypertension. **Table S12. **Association between dietary pattern overalland site-specific cancers by cardiovascular diseases.** Table S13. **Associations ofthe dietary patterns with overall and site-specific cancers separately usingobesity indicators at intermediate timepoint and metabolic syndrome componentsas response variables.** Table S14. **Associations of the dietary pattern withoverall and site-specific cancers among those with completing one or moredietary assessments.** Figure S1. **Estimated population attributable fractionfunction over 10 years for overall cancer survival time in the UK Biobankdatasettogether with point-wise 95% confidence intervals.** Figure S2. **Factor loadings for obesity-related dietary patterncalculated by using reduced rank regression using obesity indicators atintermediate timepoint as response variables.** Figure S3. **Factor loadings forobesity-related dietary pattern calculated by using reduced rank regressionusing metabolic syndrome components as response variables.** Figure S4. **Factorloadings for obesity-related dietary pattern calculated by using reduced rankregression among people with 1+ times of 24-h online dietary assessments in theUK Biobank.** Figure S5. **Factor loadings for obesity-related dietary patterncalculated by using reduced rank regression among people with 3+ times of 24-honline dietary assessments in the UK Biobank.** Figure S6. **Factorloadings for obesity-related dietary pattern calculated by using reduced rankregression among people with 4+ times of 24-h online dietary assessments in theUK Biobank.** Figure S7. **Factor loadings for obesity-related dietarypattern calculated by using reduced rank regression among people with 5 timesof 24-h online dietary assessments in the UK Biobank.

## Data Availability

The data are available on application to the UK Biobank (www.ukbiobank.ac.uk/), but restrictions apply to the availability of these data, which were used under license for the current study, and so are not publicly available. Data are however available from the authors upon reasonable request and with permissions of UK Biobank.

## Abbreviations

AIC: Akaike information criterion
BMI: Body mass index
CI: Confidence intervals
CVD: Cardiovascular diseases
DP: Dietary pattern
HbA1c: Glycated hemoglobin A1c
HDL: High-density lipoprotein
HR: Hazard ratio
HRT: Hormone replacement therapy
ICD: International Classification of Diseases
LDL: Low-density lipoprotein
PAF: Population-attributable fraction
MetS: Metabolic syndrome
MET: Metabolic equivalent
RRR: Reduced-rank regression
SD: Standard deviation
WHR: Waist-to-hip ratio
