# Supplementary material for: Associations between an obesity-related dietary pattern and incidence of overall and site-specific cancers: a prospective cohort study
Source: BMC Med. 2023 Jul 10;21:251. doi: 10.1186/s12916-023-02955-y (PMC10332028; doi:10.1186/s12916-023-02955-y)
Supplement: Supplementary file 1 — Additional file 1: Table S1. The contents of food groups. TableS2. ICD-10 code of 19 types of cancer. Table S3. Missing values and proportionsof covariates. Table S4. Explained variationin response variables for eachobesity-related dietary pattern as assessed using reduced rank regression andcorrelation coefficient between obesity-related dietary patterns and responsevariables. Table S5. Multivariable HRs and 95% CIs of the effect of dietarypattern on total and 19 site-specific cancers from, complete case analysis,excluding first 2 years incident cancer case, only including participants with5 times dietary assessments, and considering competing risk of non-cancerdeath. Table S6. Association between dietary pattern overall and site-specificcancers by sex. Table S7. Association between dietary pattern overall and site-specificcancers by age group. Table S8. Association between dietary pattern overall andsite-specific cancers by smoking status. Table S9. Association between dietarypattern overall and site-specific cancers by physical activity. Table S10. Association between dietary pattern overall and site-specific cancers bydiabetes. Table S11. Association between dietary pattern overall and site-specificcancers by hypertension. Table S12. Association between dietary pattern overalland site-specific cancers by cardiovascular diseases. Table S13. Associations ofthe dietary patterns with overall and site-specific cancers separately usingobesity indicators at intermediate timepoint and metabolic syndrome componentsas response variables. Table S14. Associations of the dietary pattern withoverall and site-specific cancers among those with completing one or moredietary assessments. Figure S1. Estimated population attributable fractionfunction over 10 years for overall cancer survival time in the UK Biobankdatasettogether with point-wise 95% confidence intervals. Figure S2. Factor loadings for obesity-related dietary patterncalculated by using reduced rank regression using o [file 12916_2023_2955_MOESM1_ESM.docx]

**Associations between an obesity-related dietary pattern and incidence of overall and site-specific cancers: a prospective cohort study**

***SUPPLEMENTARY INFORMATION***

Maiwulamujiang Maimaitiyiming, Hongxi Yang, Lihui Zhou, Xinyu Zhang, Qiliang Cai, Yaogang Wang

Procedure of setting BMI and WHR measured in 2014 as response variables to derive obesity-related DP using RRR is as follows: First, we excluded those (n=2934) with incident cancers or censoring before 2015-01-01 from study participants (N=11,4289); Next, we used individuals (17,457) who took anthropometric measurements in 2014 to derive obesity-related DPs. RRR produce in SAS can automatically calculate dietary score for those without response variable values based on RRR coefficients estimated from other observations with complete data. Therefore, those without response variable values also included in analyses to improve statistical power. Finally, there were 110,201 participants included in analyses.

**Table S1** The contents of food groups

| **Food groups** | **Food items** |
| --- | --- |
| **Pasta, rice and cereals** | White pasta, White rice, Couscous, Other grain |
| **Whole meal pasta, rice and cereals** | Brown rice, Whole meal pasta |
| **High fibre breakfast cereals** | Whole-wheat cereal, Bran cereal, Porridge, Muesli, Oat crunch, Oatcakes |
| **Other breakfast cereals** | Other cereal, Plain cereal, Sweetened cereal |
| **Cheese** | Hard cheese, soft cheese, Goat's cheese, blue cheese, Feta, Mozzarella, Cheese spread, other cheese |
| **Low fat cheese** | Low fat hard cheese, Low fat cheese spread, Cottage cheese |
| **Ice cream, cream and dairy desserts** | Ice-cream, Other milk-based pudding, Yorkshire pudding, Milk-based pudding, Cheesecake |
| **Liver** | Liver of animals |
| **Red meat** | Pork, Beef, Lamb, Other meat |
| **Poultry** | Poultry |
| **Bacon and ham** | Ham, Bacon, Sausage |
| **Oily fish** | Oily fish |
| **Other fish** | White fish, other fish, Tinned tuna |
| **Coated or breaded meat and fish** | Breaded fish, battered fish, Crumbed or deep-fried poultry, Sushi |
| **Eggs and eggs dishes** | Whole egg, Omelets, Eggs in sandwiches, Scotch egg, other egg |
| **Seafood** | Lobster/crab, Shellfish, Prawns |
| **Meat alternatives** | Vegetarian sausages/burgers, Other vegetarian alternative, Tofu, Quorn |
| **Fresh vegetable** | Vegetable pieces, Coleslaw, Side salad, Avocado, Beetroot, Broccoli, Butternut squash, Cabbage/kale, Carrot, Cauliflower, Celery, Courgetti, Cucumber, Garlic, Leek, Lettuce, Mushroom, Onion, Parsnip, Sweet pepper, Spinach, Sprouts, Sweet potato, Fresh tomato, Tinned tomato, Turnip/swede, Watercress, Other vegetables, Sweetcorn, Olives |
| **Frozen vegetable** | Mixed vegetable |
| **Legumes** | Broad bean, green bean, Pea, Baked bean, Pulses |
| **Boiled and baked potato** | Boiled/baked potatoes, Mashed potato |
| **Fruits** | Prune, Dried fruit, Mixed fruit, Apple, Banana, Berry, Cherry, Grapefruit, Grape, Mango, Melon, Orange, Satsuma, Peach/nectarine, Pear, Pineapple, Plum, Other fruit |
| **Soups** | Powdered/instant soup, Canned soup, Homemade soup |
| **Nuts and seeds** | Unsalted peanuts, Unsalted nuts, Seeds |
| **Crisps, chips and savory snacks** | Pizza, Crisp, Fried potatoes, Cheesy biscuits, Salted nuts, Salted peanuts, Crispbread, Indian snacks, Savory biscuits, Another savory snack |
| **Buns, cakes, pastries and fruit pies, puddings, biscuits** | Fruitcake, Double crust pastry, Single crust pastry, Scotch pancake, Scone, Sponge pudding, Danish pastry, Doughnut, Pancake, sweet biscuits, Chocolate-covered biscuits, Other dessert |
| **Sugar added to food or drink** | Sugar added to cereal, Sugar added to coffee, Sugar added to tea |
| **Artificial sweetener added to food or drink** | Artificial sweetener added to tea, Artificial sweetener added to cereal, Artificial sweetener added to coffee, |
| **Sugar, preserves and confectionery** | Diet sweets, Stewed fruit, Dark chocolate, Milk chocolate, other sweets, Chocolate-covered raisin, Chocolate bar, Chocolate biscuits, Chocolate sweet, White chocolate, Sweets, |
| **Fruit juice** | Orange juice, Grapefruit juice, Pure fruit/vegetable juice, Fruit smoothie, Squash |
| **High sugar beverages** | Fizzy drink, Hot chocolate, Dairy smoothie, Low calorie drink, Low calorie hot chocolate, other drink |
| **Tea** | Standard tea, Rooibos tea, green tea, Herbal tea, other tea, |
| **Instant coffee** | Instant coffee |
| **Other Coffee** | Espresso, Cappuccino, Filtered coffee, Latte, |
| **Water** | Drinking water |
| **Spirits and liqueurs** | Spirits, Other alcohol, |
| **Wine** | Red wine, Rose wine, White wine, Fortified wine, |
| **Beer and cider** | Beer and cider |
| **Whole milk** | Whole milk >3.6g fat per 100g |
| **Skimmed milk** | Skimmed milk and semi skimmed milk >1g fat per 100g |
| **Other milk** | Rice milk, oat milk and soy milk |
| **Whole meal bread** | Whole meal sliced bread, whole meal baguette, whole meal bap, whole meal bread roll, mixed sliced bread, seeded sliced bread, mixed baguette, seeded baguette, mixed bag, seeded bap, mixed roll, seeded roll |
| **White bread** | Sliced bread, baguette, bap, bread roll and other bread |
| **Olive oil used in cooking** | Olive oil used in cooking |
| **Low and normal fat butter** | Low and normal fat butter on bread/crackers |
| **Plant-based margarine** | Low and normal fat plant-based margarine |
| **polyunsaturated margarine** | Low and normal fat polyunsaturated margarine |

**Table S2** ICD-10 code of 19 types of cancer

| **Cancer type/sites** | **ICD-10 code** |
| --- | --- |
| **Oral** | C00, C01, C02, C03, C04, C05, C06 |
| **Esophagus** | C15 |
| **Stomach** | C16 |
| **Colorectal** | C18, C19, C20 |
| **Liver** | C22 |
| **Pancreas** | C25 |
| **Lung** | C34 |
| **Malignant** **melanoma** | C43 |
| **Breast** | C50 |
| **Cervix** | C53 |
| **Endometrium** | C54 |
| **Ovary** | C56 |
| **Prostate** | C61 |
| **Kidney** | C64, C65 |
| **Bladder** | C67 |
| **Thyroid** | C73 |
| **Non-Hodgkin lymphoma** | C82, C83, C84, C85 |
| **Multiple** **myeloma** | C90 |
| **Leukemia** | C91, C92, C93, C94, C95 |

Table S3 Missing values and proportions of covariates

| Variables | Missing number  and proportion |
| --- | --- |
| Ethnicity | 38 (0.03%) |
| Physical activity | 16019 (14.02%) |
| Smoking status | 243 (0.21%) |
| Energy intake | 2 (<0.01%) |
| Townsend deprivation index | 136 (0.12%) |
| Hypertension | 141 (0.12%) |
| Diabetes | 38 (0.03%) |
| Oral contraceptive use | 110 (0.18%) |
| Hormone replacement therapy | 140 (0.22%) |
| Body mass index | 1526 (1.34%) |
| Waist-to-hip ratio | 150 (0.13%) |

BMI, body mass index

**Table S4** Explained variation (%) in response variables for each obesity-related dietary pattern as assessed using reduced rank regression and correlation coefficient between obesity-related dietary patterns and response variables

| **Dietary pattern** | **Explained variation (%)** | | | **Correlation coefficient** | |
| --- | --- | --- | --- | --- | --- |
|  | **BMI** | **WHR** | **Total** | **BMI** | **WHR** |
| **1** | 8.09 | 14.22 | 11.16 | 0.60 | 0.80 |
| **2** | 3.28 | 1.87 | 2.57 | 0.80 | -0.60 |

BMI, body mass index; WHR: Waist-to-hip ratio.

**Table S5** Multivariable HRs and 95% CIs of the effect of dietary pattern on total and 20 site-specific cancers from complete case analysis, excluding first 2 years incident cancer case, only including participants with 5 times dietary assessments, and considering competing risk of non-cancer death

| **Type of cancer** | **Complete case analysis** | | **Excluding first 2 years** | | **5 times dietary assessments** | | **Competing risk model** | |
| --- | --- | --- | --- | --- | --- | --- | --- | --- |
|  | **HR (95% CI)** | **Corrected *P*-value** | **HR (95% CI)** | **Corrected *P*-value** | **HR (95% CI)** | **Corrected *P*-value** | **HR (95% CI)** | **Corrected *P*-value** |
| Overall | 1.04 (1.02, 1.05) | <0.001 | 1.04 (1.03, 1.06) | <0.001 | 1.12 (1.07, 1.17) | <0.001 | 1.03 (1.02, 1.04) | <0.001 |
| Oral | 1.34 (1.11, 1.61) | 0.005 | 1.35 (1.11, 1.65) | 0.009 | 0.62 (0.23, 1.66) | 0.420 | 1.33 (1.16, 1.53) | <0.001 |
| Esophagus | 1.19 (1.10, 1.30) | <0.001 | 1.23 (1.12, 1.34) | <0.001 | 1.54 (1.21, 1.95) | <0.001 | 1.18 (1.08, 1.30) | <0.001 |
| Stomach | 1.10 (1.00, 1.22) | 0.078 | 1.09 (0.98, 1.22) | 0.182 | 1.59 (1.19, 2.14) | 0.008 | 1.04 (0.93, 1.15) | 0.620 |
| Colorectal | 1.10 (1.06, 1.15) | <0.001 | 1.10 (1.06, 1.15) | <0.001 | 1.09 (0.96, 1.25) | 0.299 | 1.08 (1.05, 1.12) | <0.001 |
| Liver | 1.19 (1.07, 1.33) | 0.005 | 1.17 (1.05, 1.30) | 0.011 | 2.13 (1.56, 2.91) | <0.001 | 1.18 (1.07, 1.30) | 0.003 |
| Pancreas | 1.11 (1.02, 1.20) | 0.030 | 1.02 (0.93, 1.11) | 0.736 | 1.12 (0.81, 1.56) | 0.508 | 1.04 (0.96, 1.12) | 0.429 |
| Lung | 1.11 (1.06, 1.18) | <0.001 | 1.13 (1.07, 1.19) | <0.001 | 1.34 (1.10, 1.62) | 0.011 | 1.12 (1.07, 1.18) | <0.001 |
| Malignant Melanoma | 0.92 (0.86, 0.98) | 0.017 | 0.93 (0.87, 1.00) | 0.058 | 0.91 (0.73, 1.14) | 0.469 | 0.93 (0.87, 0.98) | 0.017 |
| Premenopausal Breast | 0.93 (0.88, 0.99) | 0.029 | 0.94 (0.89, 0.99) | 0.052 | 0.78 (0.59, 1.02) | 0.137 | 0.97 (0.92, 1.02) | 0.400 |
| Postmenopausal Breast | 1.00 (0.95, 1.05) | 0.993 | 1.02 (0.98, 1.08) | 0.410 | 0.86 (0.74, 1.01) | 0.137 | 1.00 (0.96, 1.05) | 0.971 |
| Cervix | 1.33 (1.02, 1.74) | 0.053 | 1.04 (0.77, 1.40) | 0.823 | 0.00 (0.00, Inf) | 0.979 | 1.17 (0.88, 1.55) | 0.400 |
| Endometrium | 1.27 (1.16, 1.40) | <0.001 | 1.25 (1.13, 1.37) | <0.001 | 1.15 (0.82, 1.61) | 0.469 | 1.20 (1.10, 1.31) | <0.001 |
| Ovary | 1.10 (0.99, 1.23) | 0.106 | 1.13 (1.01, 1.27) | 0.058 | 0.79 (0.54, 1.16) | 0.304 | 1.07 (0.97, 1.19) | 0.318 |
| Prostate | 0.98 (0.96, 1.01) | 0.341 | 0.98 (0.96, 1.01) | 0.347 | 1.06 (0.97, 1.16) | 0.299 | 0.97 (0.94, 0.99) | 0.026 |
| Kidney | 1.15 (1.07, 1.24) | <0.001 | 1.09 (1.01, 1.18) | 0.052 | 1.29 (1.03, 1.62) | 0.081 | 1.11 (1.04, 1.18) | 0.004 |
| Bladder | 1.08 (1.01, 1.14) | 0.032 | 1.09 (1.02, 1.16) | 0.016 | 1.12 (0.93, 1.35) | 0.304 | 1.11 (1.04, 1.17) | 0.003 |
| Thyroid | 1.31 (1.10, 1.55) | 0.005 | 1.35 (1.15, 1.58) | <0.001 | 1.92 (1.36, 2.71) | <0.001 | 1.27 (1.09, 1.48) | 0.004 |
| Non-Hodgkin lymphoma | 0.97 (0.91, 1.04) | 0.427 | 0.99 (0.92, 1.06) | 0.736 | 1.20 (0.99, 1.46) | 0.137 | 0.99 (0.94, 1.05) | 0.800 |
| Multiple Myeloma | 1.01 (0.92, 1.11) | 0.923 | 1.02 (0.93, 1.13) | 0.736 | 0.73 (0.52, 1.03) | 0.137 | 1.00 (0.92, 1.09) | 0.963 |
| Leukemia | 0.97 (0.89, 1.05) | 0.427 | 1.05 (0.96, 1.14) | 0.356 | 1.14 (0.92, 1.41) | 0.304 | 1.02 (0.95, 1.10) | 0.690 |

All models were stratified by sex and study region and adjusted for age, ethnicity, Townsend deprivation index, education attainment, physical activity, smoking status, and total energy intake per day (log-transformed).

**Table S6** Associations of obesity-related dietary pattern with overall and site-specific cancers by sex

|  | Male | | Female | | Corrected p for interaction |
| --- | --- | --- | --- | --- | --- |
|  | HR (95CI%) | Corrected P-value | HR (95CI%) | Corrected P-value |  |
| Overall | 1.05 (1.03, 1.07) | <0.001 | 1.02 (1.00, 1.04) | 0.177 | 0.764 |
| Oral | 1.43 (1.16, 1.76) | 0.003 | 1.30 (0.96, 1.77) | 0.252 | 0.804 |
| Esophagus | 1.24 (1.13, 1.36) | <0.001 | 1.06 (0.89, 1.26) | 0.558 | 0.486 |
| Stomach | 1.12 (1.01, 1.24) | 0.071 | 0.78 (0.61, 0.98) | 0.133 | 0.195 |
| Colorectal | 1.14 (1.09, 1.19) | <0.001 | 0.98 (0.92, 1.05) | 0.604 | 0.001 |
| Liver | 1.09 (0.97, 1.23) | 0.197 | 1.37 (1.14, 1.65) | 0.005 | 0.354 |
| Pancreas | 1.05 (0.95, 1.16) | 0.369 | 1.05 (0.92, 1.19) | 0.558 | 0.804 |
| Lung | 1.21 (1.13, 1.28) | <0.001 | 1.05 (0.97, 1.14) | 0.358 | 0.248 |
| Malignant Melanoma | 0.93 (0.86, 1.01) | 0.124 | 0.94 (0.86, 1.03) | 0.345 | 0.764 |
| Premenopausal Breast | NA | NA | 0.97 (0.92, 1.02) | 0.403 | NA |
| Postmenopausal Breast | NA | NA | 1.00 (0.96, 1.05) | 0.883 | NA |
| Cervix | NA | NA | 1.18 (0.92, 1.51) | 0.345 | NA |
| Endometrium | NA | NA | 1.22 (1.12, 1.32) | <0.001 | NA |
| Ovary | NA | NA | 1.08 (0.98, 1.20) | 0.311 | NA |
| Prostate | 0.97 (0.95, 1.00) | 0.077 | NA | NA | NA |
| Kidney | 1.12 (1.03, 1.21) | 0.016 | 1.06 (0.91, 1.22) | 0.558 | 0.354 |
| Bladder | 1.15 (1.08, 1.22) | <0.001 | 0.94 (0.82, 1.07) | 0.463 | 0.012 |
| Thyroid | 1.02 (0.80, 1.31) | 0.871 | 1.46 (1.21, 1.76) | <0.001 | 0.230 |
| Non-Hodgkin lymphoma | 1.01 (0.94, 1.09) | 0.827 | 0.93 (0.84, 1.04) | 0.345 | 0.201 |
| Multiple Myeloma | 0.88 (0.78, 0.99) | 0.071 | 1.29 (1.13, 1.49) | <0.001 | <0.001 |
| Leukemia | 1.03 (0.95, 1.12) | 0.554 | 1.06 (0.91, 1.22) | 0.558 | 0.512 |

NA represent the missing parameters due to sex-specific cancer sites. All models were stratified by sex and study region and adjusted for age, ethnicity, Townsend deprivation index, education attainment, physical activity, smoking status, and total energy intake per day (log-transformed).

**Table S7** Associations of obesity-related dietary pattern with overall and site-specific cancers by age group

|  | Age>=65 | | Age<65 | | Corrected *p* for interaction |
| --- | --- | --- | --- | --- | --- |
|  | HR (95CI%) | Corrected *P*-value | HR (95CI%) | Corrected *P*-value |  |
| Overall | 1.04 (1.02, 1.07) | 0.007 | 1.03 (1.02, 1.05) | <0.001 | <0.001 |
| Oral | 1.89 (1.20, 2.97) | 0.018 | 1.28 (1.06, 1.54) | 0.021 | 0.273 |
| Esophagus | 1.01 (0.86, 1.17) | 0.985 | 1.28 (1.16, 1.41) | <0.001 | 0.292 |
| Stomach | 0.80 (0.65, 0.97) | 0.066 | 1.14 (1.02, 1.27) | 0.048 | 0.159 |
| Colorectal | 1.11 (1.04, 1.19) | 0.011 | 1.08 (1.03, 1.12) | 0.004 | 0.720 |
| Liver | 1.07 (0.86, 1.33) | 0.750 | 1.21 (1.08, 1.36) | 0.004 | 0.318 |
| Pancreas | 1.04 (0.89, 1.22) | 0.750 | 1.05 (0.96, 1.15) | 0.425 | 0.720 |
| Lung | 1.09 (1.00, 1.20) | 0.122 | 1.15 (1.08, 1.21) | 0.000 | 0.827 |
| Malignant Melanoma | 0.83 (0.73, 0.95) | 0.018 | 0.96 (0.90, 1.02) | 0.349 | 0.537 |
| Premenopausal Breast | 0.82 (0.60, 1.12) | 0.368 | 0.97 (0.93, 1.03) | 0.425 | 0.659 |
| Postmenopausal Breast | 1.06 (0.95, 1.18) | 0.426 | 0.99 (0.95, 1.04) | 0.832 | 0.318 |
| Cervix | 2.40 (1.36, 4.26) | 0.013 | 1.01 (0.77, 1.33) | 0.938 | 0.095 |
| Endometrium | 1.46 (1.21, 1.76) | <0.001 | 1.16 (1.06, 1.27) | 0.007 | 0.329 |
| Ovary | 1.00 (0.75, 1.34) | 0.989 | 1.10 (0.98, 1.22) | 0.166 | 0.880 |
| Prostate | 1.00 (0.95, 1.06) | 0.953 | 0.97 (0.94, 1.00) | 0.048 | 0.095 |
| Kidney | 1.08 (0.93, 1.25) | 0.440 | 1.12 (1.03, 1.21) | 0.021 | 0.273 |
| Bladder | 1.28 (1.17, 1.41) | <0.001 | 1.04 (0.97, 1.11) | 0.425 | 0.003 |
| Thyroid | 1.45 (0.98, 2.14) | 0.124 | 1.25 (1.07, 1.47) | 0.018 | 0.720 |
| Non-Hodgkin lymphoma | 0.88 (0.78, 1.00) | 0.122 | 1.03 (0.96, 1.11) | 0.479 | 0.547 |
| Multiple Myeloma | 0.95 (0.79, 1.14) | 0.750 | 1.04 (0.93, 1.15) | 0.556 | 0.720 |
| Leukemia | 1.02 (0.90, 1.16) | 0.898 | 1.04 (0.95, 1.14) | 0.481 | 0.827 |

All models were stratified by sex and study region and adjusted for age, ethnicity, Townsend deprivation index, education attainment, physical activity, smoking status, and total energy intake per day (log-transformed).

**Table S8** Associations of obesity-related dietary pattern with overall and site-specific cancers by smoking status

|  | Current | | Previous | | Never | | Corrected *p* for interaction |
| --- | --- | --- | --- | --- | --- | --- | --- |
|  | HR (95CI%) | Corrected *P*-value | HR (95CI%) | Corrected *P*-value | HR (95CI%) | Corrected *P*-value |  |
| Overall | 1.05 (1.01, 1.09) | 0.056 | 1.02 (1.00, 1.04) | 0.121 | 1.05 (1.03, 1.07) | <0.001 | 0.550 |
| Oral | 1.39 (0.97, 1.98) | 0.177 | 1.12 (0.86, 1.45) | 0.553 | 1.66 (1.24, 2.23) | 0.004 | 0.187 |
| Esophagus | 1.43 (1.18, 1.74) | <0.001 | 1.14 (1.02, 1.28) | 0.066 | 1.14 (0.99, 1.32) | 0.123 | 0.283 |
| Stomach | 1.04 (0.75, 1.45) | 0.891 | 0.96 (0.84, 1.10) | 0.657 | 1.17 (1.00, 1.37) | 0.088 | 0.212 |
| Colorectal | 1.04 (0.93, 1.17) | 0.640 | 1.07 (1.02, 1.13) | 0.037 | 1.12 (1.06, 1.19) | <0.001 | 0.796 |
| Liver | 1.15 (0.88, 1.50) | 0.475 | 1.11 (0.96, 1.29) | 0.304 | 1.25 (1.06, 1.47) | 0.016 | 0.071 |
| Pancreas | 0.99 (0.82, 1.20) | 0.908 | 1.29 (1.15, 1.44) | <0.001 | 0.86 (0.76, 0.97) | 0.036 | <0.001 |
| Lung | 1.08 (0.99, 1.18) | 0.200 | 1.18 (1.11, 1.26) | <0.001 | 1.05 (0.92, 1.19) | 0.542 | 0.152 |
| Malignant Melanoma | 0.96 (0.73, 1.26) | 0.891 | 0.93 (0.85, 1.02) | 0.242 | 0.93 (0.86, 1.01) | 0.126 | 0.550 |
| Premenopausal Breast | 0.99 (0.84, 1.16) | 0.908 | 0.97 (0.89, 1.06) | 0.657 | 0.97 (0.91, 1.04) | 0.542 | 0.885 |
| Postmenopausal Breast | 0.87 (0.74, 1.01) | 0.177 | 1.03 (0.96, 1.10) | 0.553 | 1.00 (0.95, 1.07) | 0.931 | 0.237 |
| Cervix | 0.03 (0.00, 0.19) | <0.001 | 1.33 (0.93, 1.90) | 0.242 | 1.20 (0.85, 1.70) | 0.385 | 0.052 |
| Endometrium | 2.53 (1.77, 3.62) | <0.001 | 1.02 (0.88, 1.18) | 0.822 | 1.27 (1.14, 1.42) | <0.001 | 0.017 |
| Ovary | 0.78 (0.50, 1.23) | 0.475 | 0.96 (0.81, 1.13) | 0.668 | 1.20 (1.05, 1.37) | 0.016 | 0.152 |
| Prostate | 0.86 (0.78, 0.94) | 0.008 | 0.95 (0.92, 0.99) | 0.042 | 1.02 (0.98, 1.06) | 0.349 | 0.021 |
| Kidney | 1.19 (1.02, 1.40) | 0.087 | 0.99 (0.89, 1.10) | 0.850 | 1.23 (1.09, 1.38) | 0.004 | 0.059 |
| Bladder | 1.49 (1.30, 1.70) | <0.001 | 1.16 (1.08, 1.25) | <0.001 | 0.86 (0.78, 0.96) | 0.016 | <0.001 |
| Thyroid | 1.35 (0.91, 2.00) | 0.258 | 0.87 (0.66, 1.16) | 0.544 | 1.55 (1.28, 1.87) | <0.001 | 0.071 |
| Non-Hodgkin lymphoma | 0.92 (0.73, 1.16) | 0.640 | 0.96 (0.87, 1.06) | 0.553 | 1.03 (0.95, 1.13) | 0.542 | 0.550 |
| Multiple Myeloma | 0.86 (0.57, 1.30) | 0.640 | 1.16 (1.02, 1.32) | 0.078 | 0.91 (0.80, 1.03) | 0.220 | 0.152 |
| Leukemia | 0.94 (0.73, 1.21) | 0.799 | 1.05 (0.95, 1.17) | 0.544 | 1.01 (0.90, 1.13) | 0.931 | 0.173 |

All models were stratified by sex and study region and adjusted for age, ethnicity, Townsend deprivation index, education attainment, physical activity, smoking status, and total energy intake per day (log-transformed).

**Table S9** Association of dietary pattern with overall and site-specific cancers by physical activity

|  | High | | Moderate | | Low | | Corrected *p* for interaction |
| --- | --- | --- | --- | --- | --- | --- | --- |
|  | HR (95CI%) | Corrected *P*-value | HR (95CI%) | Corrected *P*-value | HR (95CI%) | Corrected *P*-value |  |
| Overall | 1.05 (1.02, 1.07) | <0.001 | 1.02 (1.00, 1.04) | 0.220 | 1.04 (1.01, 1.07) | 0.068 | 0.156 |
| Oral | 1.47 (1.14, 1.89) | 0.008 | 1.25 (0.96, 1.64) | 0.300 | 1.28 (0.79, 2.06) | 0.445 | 0.892 |
| Esophagus | 1.28 (1.11, 1.46) | <0.001 | 1.10 (0.96, 1.25) | 0.370 | 1.23 (1.04, 1.44) | 0.068 | 0.518 |
| Stomach | 1.19 (1.02, 1.38) | 0.053 | 0.95 (0.81, 1.12) | 0.831 | 0.93 (0.76, 1.13) | 0.593 | 0.020 |
| Colorectal | 1.12 (1.06, 1.19) | <0.001 | 1.05 (1.00, 1.12) | 0.224 | 1.09 (1.01, 1.17) | 0.105 | 0.156 |
| Liver | 1.30 (1.10, 1.53) | 0.006 | 0.97 (0.82, 1.15) | 0.831 | 1.40 (1.16, 1.70) | <0.001 | 0.156 |
| Pancreas | 1.08 (0.95, 1.23) | 0.274 | 1.03 (0.91, 1.16) | 0.831 | 1.00 (0.85, 1.17) | 0.987 | 0.989 |
| Lung | 1.16 (1.08, 1.25) | <0.001 | 1.14 (1.06, 1.24) | 0.011 | 1.04 (0.93, 1.16) | 0.604 | 0.156 |
| Malignant Melanoma | 0.82 (0.74, 0.90) | <0.001 | 0.99 (0.91, 1.08) | 0.902 | 1.04 (0.92, 1.19) | 0.604 | 0.260 |
| Premenopausal Breast | 0.95 (0.87, 1.03) | 0.274 | 1.01 (0.94, 1.09) | 0.831 | 0.92 (0.82, 1.04) | 0.315 | 0.473 |
| Postmenopausal Breast | 1.06 (0.99, 1.14) | 0.146 | 0.99 (0.93, 1.05) | 0.831 | 0.93 (0.83, 1.03) | 0.286 | 0.335 |
| Cervix | 1.60 (1.12, 2.28) | 0.021 | 1.01 (0.70, 1.48) | 0.947 | 0.63 (0.30, 1.34) | 0.347 | 0.126 |
| Endometrium | 1.35 (1.17, 1.55) | <0.001 | 1.10 (0.97, 1.25) | 0.315 | 1.22 (1.01, 1.48) | 0.114 | 0.451 |
| Ovary | 1.01 (0.84, 1.20) | 0.954 | 1.10 (0.94, 1.27) | 0.439 | 1.19 (0.95, 1.48) | 0.254 | 0.518 |
| Prostate | 0.96 (0.92, 1.00) | 0.102 | 0.98 (0.94, 1.02) | 0.573 | 0.99 (0.93, 1.05) | 0.720 | 0.518 |
| Kidney | 1.12 (0.99, 1.26) | 0.124 | 1.08 (0.97, 1.19) | 0.370 | 1.19 (1.02, 1.37) | 0.101 | 0.156 |
| Bladder | 1.05 (0.96, 1.15) | 0.337 | 1.18 (1.08, 1.28) | <0.001 | 1.09 (0.96, 1.23) | 0.315 | 0.700 |
| Thyroid | 1.29 (0.99, 1.66) | 0.096 | 1.15 (0.88, 1.49) | 0.560 | 1.38 (1.08, 1.77) | 0.068 | 0.281 |
| Non-Hodgkin lymphoma | 1.11 (1.02, 1.22) | 0.048 | 0.88 (0.80, 0.97) | 0.084 | 0.98 (0.85, 1.13) | 0.856 | 0.126 |
| Multiple Myeloma | 0.97 (0.83, 1.13) | 0.710 | 1.13 (0.99, 1.28) | 0.224 | 0.80 (0.65, 1.00) | 0.129 | 0.241 |
| Leukemia | 1.10 (0.98, 1.24) | 0.125 | 1.02 (0.91, 1.14) | 0.831 | 0.86 (0.71, 1.03) | 0.222 | 0.700 |

All models were stratified by sex and study region and adjusted for age, ethnicity, Townsend deprivation index, education attainment, physical activity, smoking status, and total energy intake per day (log-transformed).

**Table S10** Associations of obesity-related dietary pattern with overall and site-specific cancers by diabetes

|  | Yes | | No | | Corrected *p* for interaction |
| --- | --- | --- | --- | --- | --- |
|  | HR (95CI%) | Corrected *P*-value | HR (95CI%) | Corrected *P*-value |  |
| Overall | 1.06 (1.00, 1.11) | 0.111 | 1.03 (1.02, 1.04) | <0.001 | 0.182 |
| Oral | 0.82 (0.31, 2.15) | 0.850 | 1.38 (1.16, 1.65) | <0.001 | 0.147 |
| Esophagus | 1.18 (0.95, 1.47) | 0.192 | 1.15 (1.05, 1.25) | 0.005 | 0.419 |
| Stomach | 1.04 (0.74, 1.46) | 0.856 | 1.03 (0.93, 1.14) | 0.658 | 0.678 |
| Colorectal | 1.16 (1.02, 1.30) | 0.084 | 1.07 (1.03, 1.11) | <0.001 | 0.419 |
| Liver | 1.02 (0.81, 1.28) | 0.865 | 1.15 (1.02, 1.28) | 0.032 | 0.686 |
| Pancreas | 1.20 (0.94, 1.55) | 0.200 | 1.01 (0.94, 1.10) | 0.760 | 0.304 |
| Lung | 0.87 (0.73, 1.04) | 0.192 | 1.15 (1.09, 1.21) | <0.001 | 0.051 |
| Malignant Melanoma | 1.28 (1.00, 1.64) | 0.119 | 0.91 (0.86, 0.97) | 0.009 | 0.113 |
| Premenopausal Breast | 1.79 (1.32, 2.44) | <0.001 | 0.96 (0.91, 1.01) | 0.128 | <0.001 |
| Postmenopausal Breast | 1.06 (0.84, 1.34) | 0.812 | 1.00 (0.96, 1.05) | 0.936 | 0.678 |
| Cervix | 16.44 (0.85, 316.70) | 0.134 | 1.13 (0.88, 1.45) | 0.465 | 0.147 |
| Endometrium | 0.70 (0.48, 1.03) | 0.135 | 1.24 (1.14, 1.35) | <0.001 | 0.147 |
| Ovary | 0.92 (0.53, 1.62) | 0.856 | 1.09 (0.98, 1.21) | 0.171 | 0.619 |
| Prostate | 1.15 (1.03, 1.29) | 0.084 | 0.97 (0.95, 1.00) | 0.096 | 0.113 |
| Kidney | 1.54 (1.14, 2.08) | 0.035 | 1.10 (1.02, 1.19) | 0.019 | 0.147 |
| Bladder | 1.18 (1.00, 1.40) | 0.119 | 1.09 (1.03, 1.16) | 0.005 | 0.147 |
| Thyroid | 0.48 (0.25, 0.91) | 0.084 | 1.33 (1.14, 1.55) | <0.001 | 0.916 |
| Non-Hodgkin lymphoma | 1.28 (0.97, 1.69) | 0.135 | 0.98 (0.92, 1.05) | 0.658 | 0.419 |
| Multiple Myeloma | 0.48 (0.29, 0.79) | 0.035 | 1.04 (0.95, 1.14) | 0.465 | 0.059 |
| Leukemia | 1.04 (0.79, 1.38) | 0.856 | 1.02 (0.95, 1.10) | 0.658 | 0.555 |

All models were stratified by sex and study region and adjusted for age, ethnicity, Townsend deprivation index, education attainment, physical activity, smoking status, and total energy intake per day (log-transformed).

**Table S11** Associations of obesity-related dietary pattern with overall and site-specific cancers by hypertension

|  | Yes | | No | | Corrected *p* for interaction |
| --- | --- | --- | --- | --- | --- |
|  | HR (95CI%) | Corrected *P*-value | HR (95CI%) | Corrected *P*-value |  |
| Overall | 1.04 (1.02, 1.06) | 0.021 | 1.02 (1.01, 1.04) | 0.011 | 0.324 |
| Oral | 1.10 (0.77, 1.56) | 0.841 | 1.43 (1.17, 1.74) | <0.001 | 0.627 |
| Esophagus | 1.10 (0.97, 1.24) | 0.359 | 1.21 (1.09, 1.35) | <0.001 | 0.851 |
| Stomach | 1.06 (0.90, 1.25) | 0.750 | 1.01 (0.90, 1.14) | 0.875 | 0.593 |
| Colorectal | 1.03 (0.97, 1.10) | 0.621 | 1.10 (1.05, 1.15) | <0.001 | 0.808 |
| Liver | 1.25 (1.07, 1.46) | 0.032 | 1.10 (0.97, 1.26) | 0.236 | 0.326 |
| Pancreas | 1.08 (0.94, 1.23) | 0.517 | 1.02 (0.92, 1.12) | 0.804 | 0.895 |
| Lung | 1.12 (1.02, 1.23) | 0.097 | 1.14 (1.08, 1.21) | <0.001 | 0.326 |
| Malignant Melanoma | 1.02 (0.92, 1.13) | 0.913 | 0.88 (0.82, 0.95) | 0.003 | 0.326 |
| Premenopausal Breast | 0.99 (0.88, 1.11) | 0.978 | 0.96 (0.91, 1.02) | 0.236 | 0.693 |
| Postmenopausal Breast | 0.99 (0.91, 1.08) | 0.978 | 1.00 (0.95, 1.05) | 0.980 | 0.895 |
| Cervix | 0.99 (0.62, 1.58) | 0.978 | 1.22 (0.91, 1.64) | 0.236 | 0.808 |
| Endometrium | 1.05 (0.90, 1.23) | 0.792 | 1.26 (1.14, 1.39) | <0.001 | 0.693 |
| Ovary | 1.00 (0.81, 1.25) | 0.978 | 1.10 (0.98, 1.24) | 0.195 | 0.700 |
| Prostate | 0.96 (0.92, 1.01) | 0.276 | 0.98 (0.95, 1.01) | 0.236 | 0.895 |
| Kidney | 1.17 (1.05, 1.30) | 0.032 | 1.04 (0.95, 1.14) | 0.479 | 0.920 |
| Bladder | 1.06 (0.97, 1.16) | 0.380 | 1.12 (1.05, 1.20) | 0.003 | 0.627 |
| Thyroid | 1.31 (1.02, 1.68) | 0.119 | 1.21 (1.01, 1.46) | 0.103 | 0.627 |
| Non-Hodgkin lymphoma | 1.09 (0.98, 1.21) | 0.276 | 0.93 (0.86, 1.00) | 0.134 | 0.063 |
| Multiple Myeloma | 0.99 (0.82, 1.18) | 0.978 | 1.03 (0.93, 1.14) | 0.637 | 0.920 |
| Leukemia | 1.21 (1.07, 1.37) | 0.021 | 0.94 (0.86, 1.03) | 0.236 | 0.143 |

All models were stratified by sex and study region and adjusted for age, ethnicity, Townsend deprivation index, education attainment, physical activity, smoking status, and total energy intake per day (log-transformed).

**Table S12** Associations of obesity-related dietary pattern with overall and site-specific cancers by cardiovascular diseases

|  | Yes | | No | | Corrected *p* for interaction |
| --- | --- | --- | --- | --- | --- |
|  | HR (95CI%) | Corrected *P*-value | HR (95CI%) | Corrected *P*-value |  |
| Overall | 1.02 (0.97, 1.07) | 0.974 | 1.04 (1.02, 1.05) | <0.001 | 0.920 |
| Oral | NA | 0.999 | 1.37 (1.16, 1.63) | <0.001 | 0.096 |
| Esophagus | 0.98 (0.75, 1.29) | 0.974 | 1.21 (1.11, 1.32) | <0.001 | 0.576 |
| Stomach | 1.09 (0.72, 1.64) | 0.974 | 1.04 (0.94, 1.15) | 0.522 | 0.388 |
| Colorectal | 0.94 (0.79, 1.11) | 0.974 | 1.10 (1.06, 1.14) | <0.001 | 0.388 |
| Liver | 0.66 (0.45, 0.95) | 0.109 | 1.25 (1.12, 1.38) | <0.001 | 0.096 |
| Pancreas | 1.05 (0.79, 1.38) | 0.974 | 1.04 (0.96, 1.13) | 0.427 | 0.729 |
| Lung | 1.20 (1.04, 1.38) | 0.063 | 1.11 (1.06, 1.17) | <0.001 | 0.576 |
| Malignant Melanoma | 1.03 (0.80, 1.31) | 0.974 | 0.92 (0.87, 0.98) | 0.019 | 0.388 |
| Premenopausal Breast | 1.02 (0.66, 1.57) | 0.974 | 0.97 (0.92, 1.02) | 0.351 | 0.576 |
| Postmenopausal Breast | 1.05 (0.82, 1.33) | 0.974 | 1.00 (0.96, 1.04) | 0.958 | 0.388 |
| Cervix | 6.89 (1.55, 30.65) | 0.063 | 1.11 (0.86, 1.43) | 0.510 | 0.388 |
| Endometrium | 1.09 (0.70, 1.71) | 0.974 | 1.22 (1.12, 1.32) | <0.001 | 0.855 |
| Ovary | 1.82 (0.76, 4.39) | 0.546 | 1.07 (0.97, 1.19) | 0.274 | 0.351 |
| Prostate | 1.21 (1.10, 1.32) | <0.001 | 0.96 (0.93, 0.99) | 0.004 | <0.001 |
| Kidney | 1.11 (0.88, 1.40) | 0.974 | 1.11 (1.03, 1.19) | 0.015 | 0.673 |
| Bladder | 0.99 (0.82, 1.20) | 0.974 | 1.12 (1.06, 1.19) | <0.001 | 0.576 |
| Thyroid | 0.20 (0.03, 1.28) | 0.312 | 1.30 (1.12, 1.51) | <0.001 | 0.025 |
| Non-Hodgkin lymphoma | 0.94 (0.75, 1.18) | 0.974 | 0.99 (0.93, 1.06) | 0.919 | 0.613 |
| Multiple Myeloma | 1.03 (0.71, 1.50) | 0.974 | 1.01 (0.92, 1.11) | 0.888 | 0.576 |
| Leukemia | 0.68 (0.53, 0.87) | 0.021 | 1.07 (0.99, 1.15) | 0.153 | 0.018 |

NA represent the missing parameters due to insufficient incident cancer cases. All models were stratified by sex and study region and adjusted for age, ethnicity, Townsend deprivation index, education attainment, physical activity, smoking status, and total energy intake per day (log-transformed).

**Table S13** Associations of the dietary patterns with overall and site-specific cancers separately using obesity indicators at intermediate timepoint and metabolic syndrome components as response variables

|  | Metabolic syndrome components | | Obesity indicators at intermediate timepoint | |
| --- | --- | --- | --- | --- |
|  | HR (95CI%) | Corrected *P*-value | HR (95CI%) | Corrected *P*-value |
| Overall | 1.03 (1.02, 1.05) | <0.001 | 1.05 (1.03, 1.07) | <0.001 |
| Oral | 1.29 (1.07, 1.54) | 0.011 | 1.38 (1.11, 1.71) | 0.007 |
| Esophagus | 1.20 (1.10, 1.30) | <0.001 | 1.22 (1.11, 1.34) | <0.001 |
| Stomach | 1.10 (1.00, 1.21) | 0.087 | 1.09 (0.96, 1.22) | 0.232 |
| Colorectal | 1.08 (1.04, 1.12) | <0.001 | 1.14 (1.09, 1.19) | <0.001 |
| Liver | 1.17 (1.06, 1.30) | 0.007 | 1.19 (1.07, 1.34) | 0.005 |
| Pancreas | 1.06 (0.98, 1.15) | 0.172 | 1.04 (0.96, 1.14) | 0.424 |
| Lung | 1.13 (1.07, 1.19) | <0.001 | 1.15 (1.09, 1.21) | <0.001 |
| Malignant Melanoma | 0.90 (0.85, 0.96) | 0.003 | 0.91 (0.85, 0.98) | 0.021 |
| Premenopausal Breast | 0.96 (0.91, 1.01) | 0.206 | 0.93 (0.88, 0.99) | 0.040 |
| Postmenopausal Breast | 0.99 (0.95, 1.04) | 0.862 | 1.05 (0.99, 1.10) | 0.140 |
| Cervix | 1.15 (0.88, 1.49) | 0.367 | 0.98 (0.71, 1.34) | 0.886 |
| Endometrium | 1.26 (1.16, 1.38) | <0.001 | 1.22 (1.10, 1.35) | <0.001 |
| Ovary | 1.12 (1.00, 1.24) | 0.074 | 1.13 (1.00, 1.28) | 0.069 |
| Prostate | 0.98 (0.95, 1.00) | 0.109 | 0.99 (0.96, 1.02) | 0.545 |
| Kidney | 1.11 (1.03, 1.19) | 0.011 | 1.10 (1.02, 1.20) | 0.031 |
| Bladder | 1.13 (1.07, 1.20) | <0.001 | 1.13 (1.06, 1.21) | <0.001 |
| Thyroid | 1.32 (1.13, 1.53) | <0.001 | 1.36 (1.15, 1.61) | <0.001 |
| Non-Hodgkin lymphoma | 1.03 (0.96, 1.09) | 0.495 | 0.99 (0.92, 1.06) | 0.789 |
| Multiple Myeloma | 1.01 (0.92, 1.10) | 0.869 | 0.96 (0.86, 1.06) | 0.464 |
| Leukemia | 1.01 (0.94, 1.09) | 0.828 | 1.08 (0.99, 1.17) | 0.140 |

All models were stratified by sex and study region and adjusted for age, ethnicity, Townsend deprivation index, education attainment, physical activity, smoking status, and total energy intake per day (log-transformed).

**Table S14** Associations of obesity-related dietary pattern with overall and site-specific cancers among those with completing one or more dietary assessments

|  | HR (95CI%) | Corrected *P*-value |
| --- | --- | --- |
| Overall | 1.04 (1.02, 1.06) | <0.001 |
| Oral | 1.39 (1.15, 1.69) | 0.003 |
| Esophagus | 1.24 (1.13, 1.37) | <0.001 |
| Stomach | 1.10 (0.98, 1.23) | 0.155 |
| Colorectal | 1.13 (1.08, 1.17) | <0.001 |
| Liver | 1.07 (0.94, 1.21) | 0.440 |
| Pancreas | 1.10 (1.01, 1.20) | 0.063 |
| Lung | 1.13 (1.07, 1.19) | <0.001 |
| Malignant Melanoma | 0.91 (0.85, 0.98) | 0.017 |
| Premenopausal Breast | 0.98 (0.92, 1.03) | 0.496 |
| Postmenopausal Breast | 1.02 (0.97, 1.07) | 0.597 |
| Cervix | 1.14 (0.88, 1.47) | 0.440 |
| Endometrium | 1.24 (1.13, 1.37) | <0.001 |
| Ovary | 1.07 (0.95, 1.20) | 0.394 |
| Prostate | 0.97 (0.94, 1.00) | 0.048 |
| Kidney | 1.13 (1.04, 1.22) | 0.007 |
| Bladder | 1.13 (1.06, 1.21) | <0.001 |
| Thyroid | 1.28 (1.09, 1.52) | 0.007 |
| Non-Hodgkin lymphoma | 1.00 (0.93, 1.07) | 0.962 |
| Multiple Myeloma | 1.00 (0.91, 1.11) | 0.962 |
| Leukemia | 1.03 (0.95, 1.12) | 0.496 |

All models were stratified by sex and study region and adjusted for age, ethnicity, Townsend deprivation index, education attainment, physical activity, smoking status, and total energy intake per day (log-transformed).


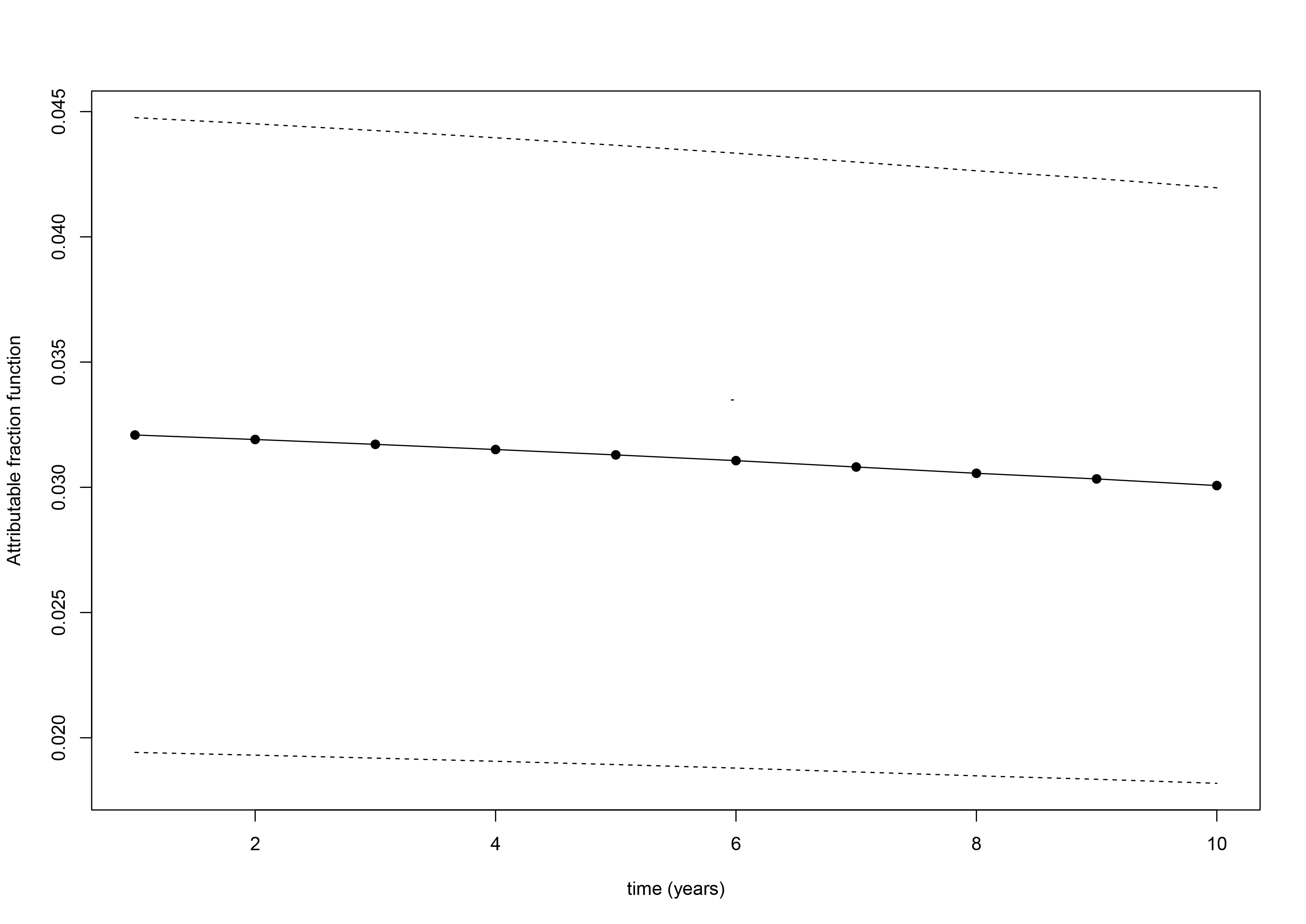


**Figure S1** Estimated Population attributable fraction function over 10 years for overall cancer survival time in the UK Biobank dataset (solid line) together with point-wise 95% confidence intervals (dashed lines)

Analysis was adjusted for sex and age, ethnicity, study region, Townsend deprivation index, education attainment, physical activity, smoking status, and total energy intake per day (log-transformed).


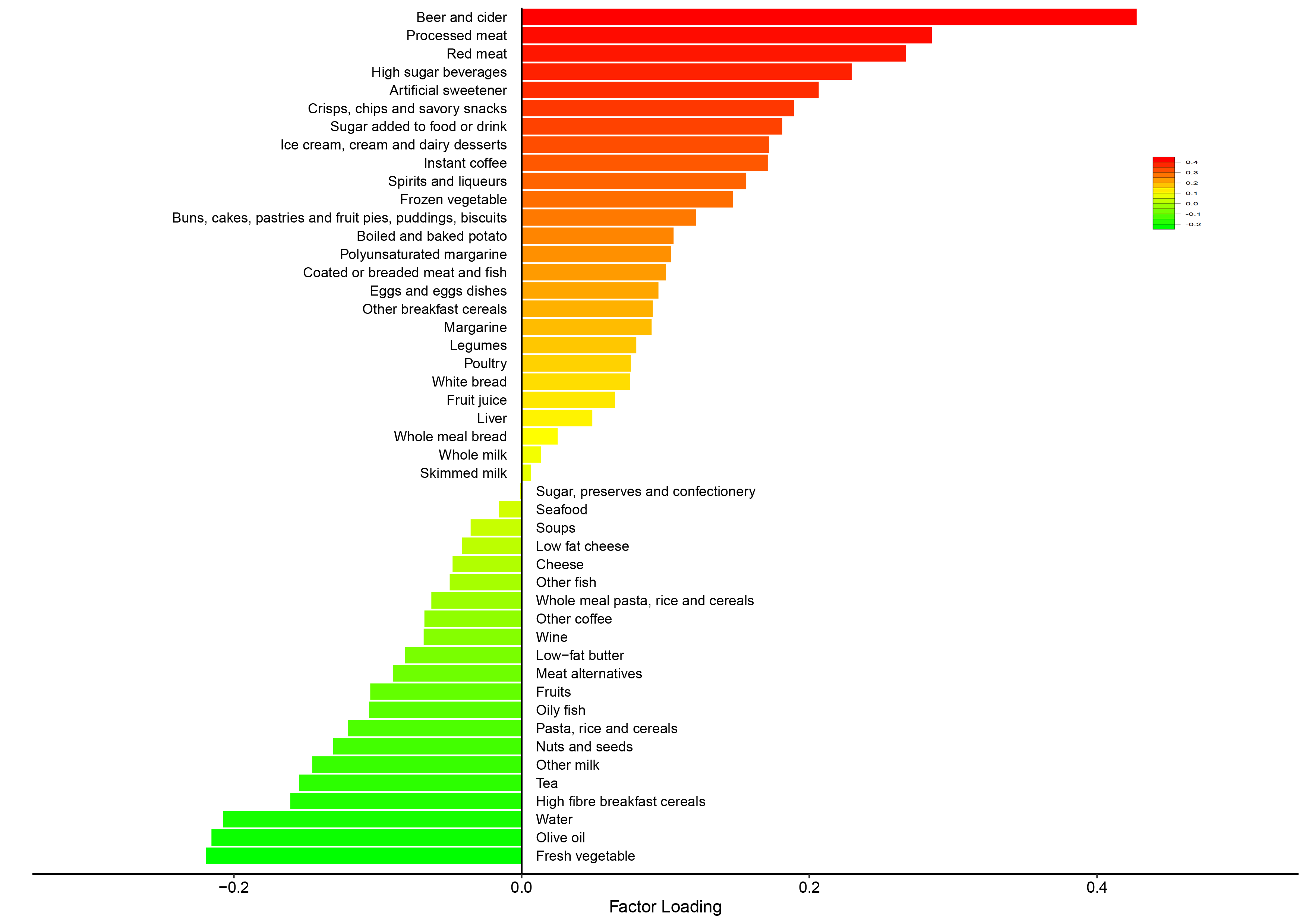


**Figure S2** Factor loadings for obesity-related dietary pattern calculated by using reduced rank regression using metabolic syndrome components as response variables


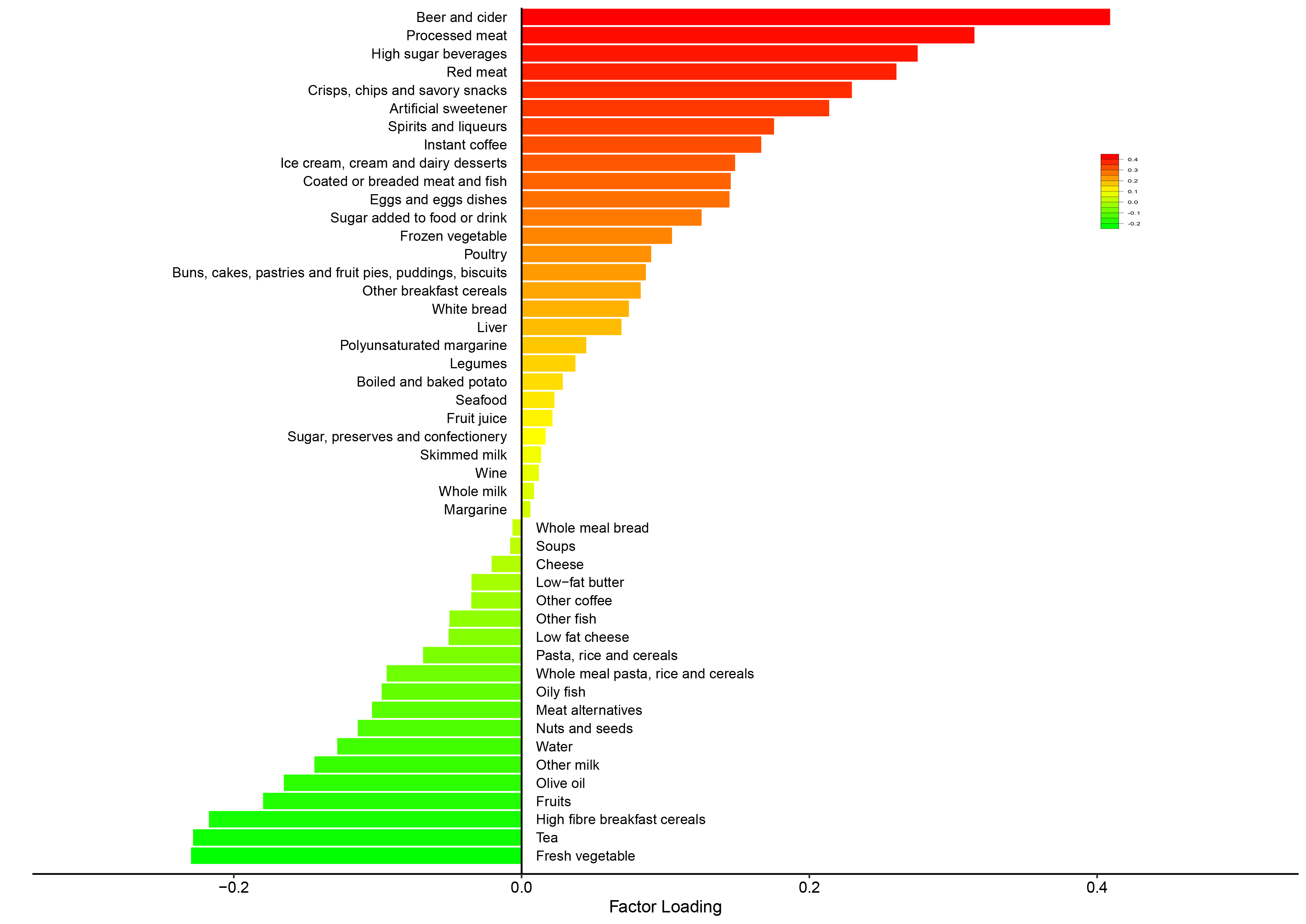


**Figure S3** Factor loadings for obesity-related dietary pattern calculated by using reduced rank regression using obesity indicators at intermediate time point as response variables


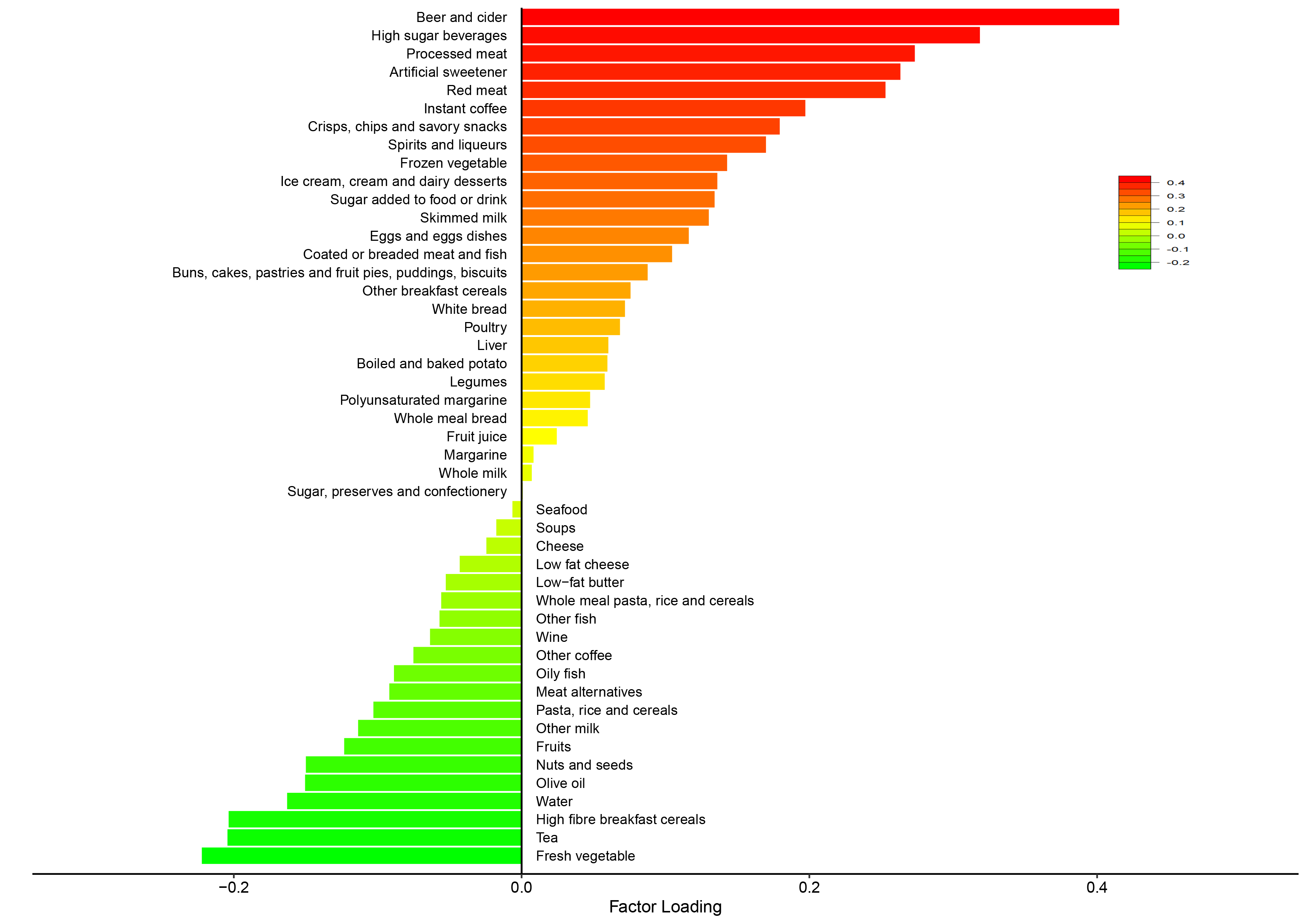


**Figure S4** Factor loadings for obesity-related dietary pattern calculated by using reduced rank regression among people with 1+ times of 24-h online dietary assessments in the UK Biobank


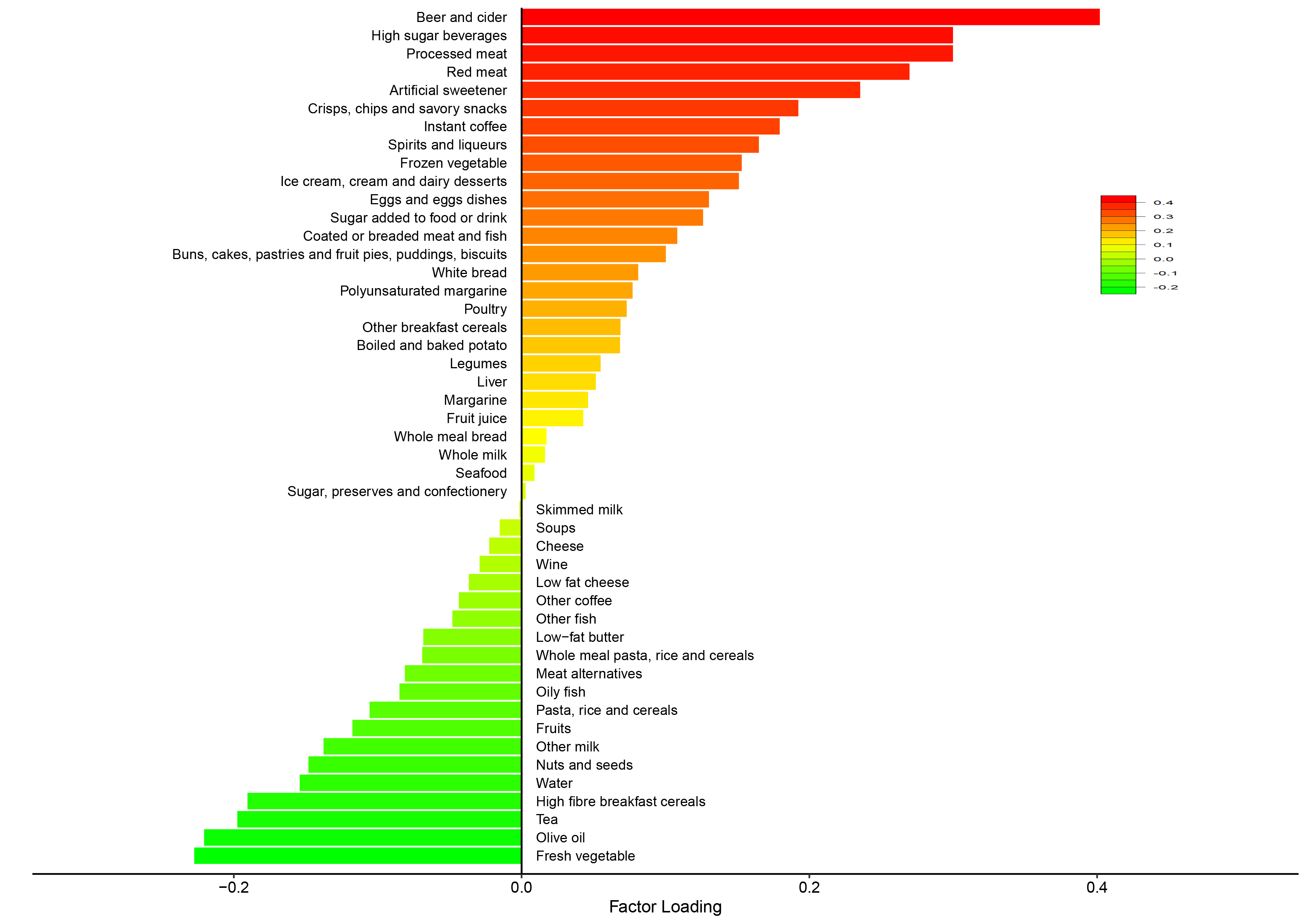


**Figure S5** Factor loadings for obesity-related dietary pattern calculated by using reduced rank regression among people with 3+ times of 24-h online dietary assessments in the UK Biobank (N=63,041)


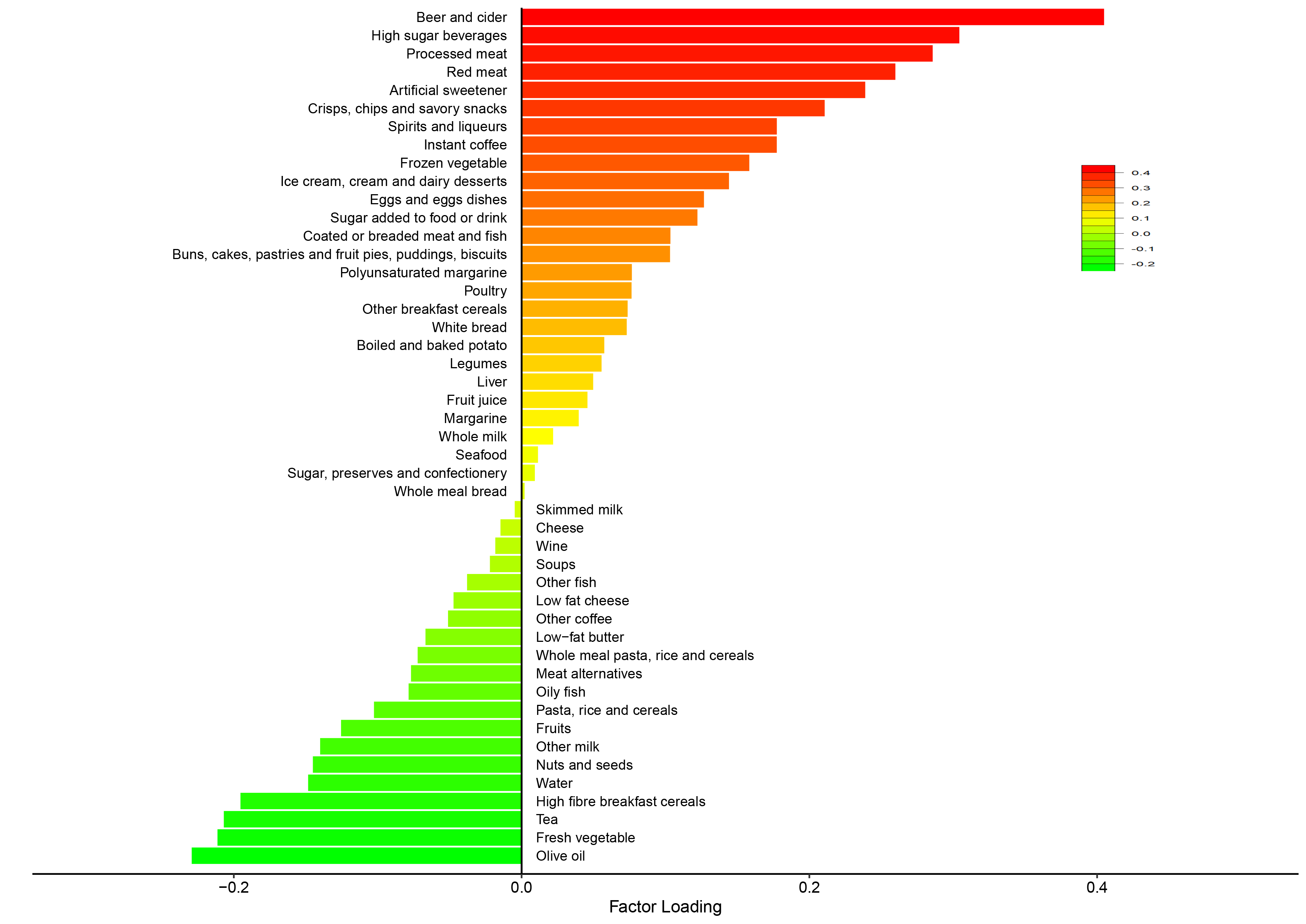


**Figure S6** Factor loadings for obesity-related dietary pattern calculated by using reduced rank regression among people with 4+ times of 24-h online dietary assessments in the UK Biobank (N=29,128)


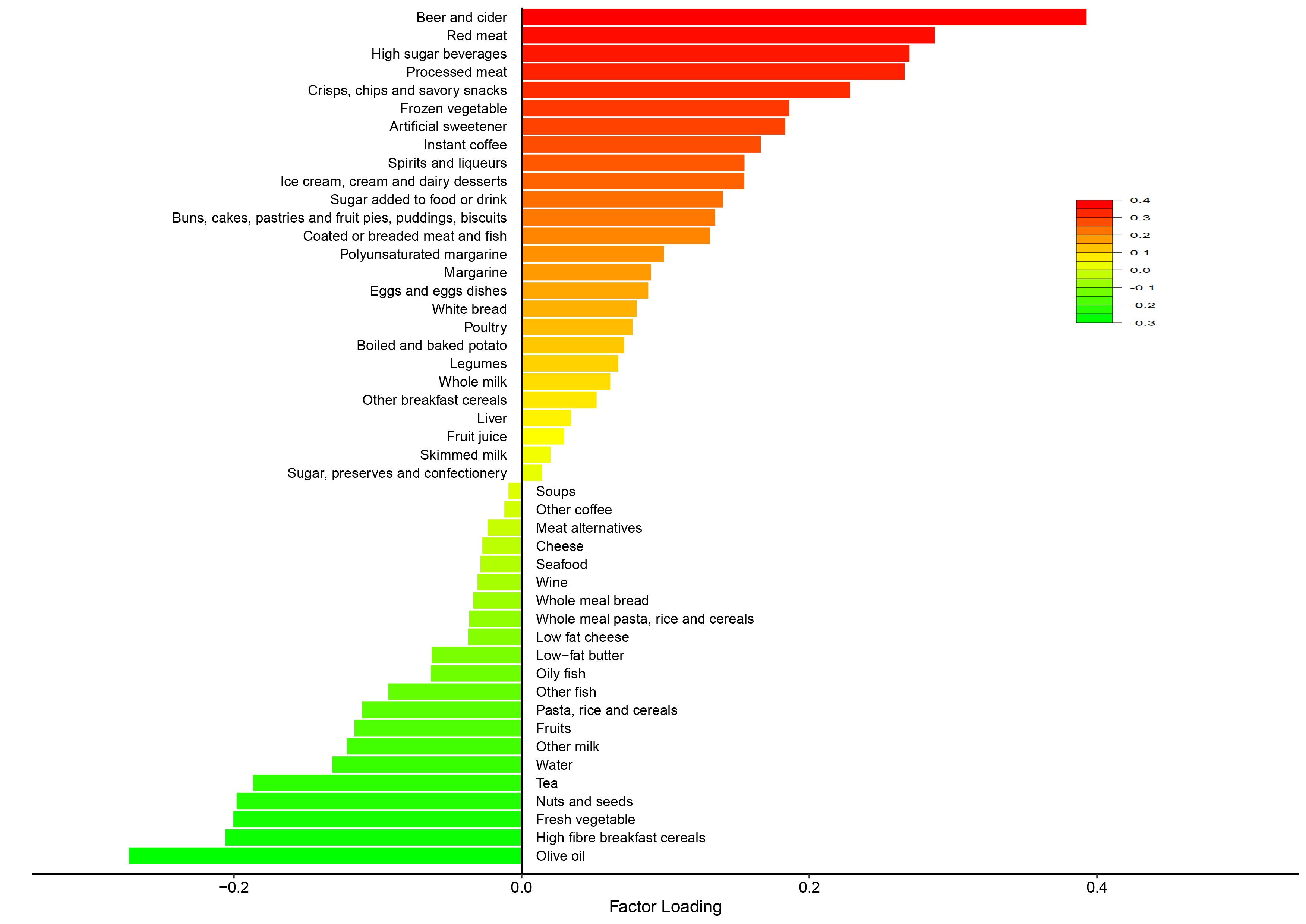


**Figure S7** Factor loadings for obesity-related dietary pattern calculated by using reduced rank regression among people with 5 times of 24-h online dietary assessments in the UK Biobank (N=4,633)
